# Analysis and Calibration of Sources of Electronic Error in PSD Sensor Response

**DOI:** 10.3390/s16050619

**Published:** 2016-04-29

**Authors:** David Rodríguez-Navarro, José Luis Lázaro-Galilea, Ignacio Bravo-Muñoz, Alfredo Gardel-Vicente, Georgios Tsirigotis

**Affiliations:** 1 Department of electronics, University of Alcalá, Alcalá de Henares (Madrid) 28801, Spain; lazaro@depeca.uah.es (J.L.L.); ibravo@depeca.uah.es (I.B.-M.); alfredo@depeca.uah.es (A.G.-V.); 2 Informatics Engineering Department, Eastern Macedonia and Thrace Institute of Technology, Kavala 65404, Greece; tsirigo@teikav.edu.gr

**Keywords:** local positioning system, PSD, infrared sensors, sensor calibration, measurement errors

## Abstract

In order to obtain very precise measurements of the position of agents located at a considerable distance using a sensor system based on position sensitive detectors (PSD), it is necessary to analyze and mitigate the factors that generate substantial errors in the system’s response. These sources of error can be divided into electronic and geometric factors. The former stem from the nature and construction of the PSD as well as the performance, tolerances and electronic response of the system, while the latter are related to the sensor’s optical system. Here, we focus solely on the electrical effects, since the study, analysis and correction of these are a prerequisite for subsequently addressing geometric errors. A simple calibration method is proposed, which considers PSD response, component tolerances, temperature variations, signal frequency used, signal to noise ratio (SNR), suboptimal operational amplifier parameters, and analog to digital converter (ADC) quantitation SNR_Q_, *etc*. Following an analysis of these effects and calibration of the sensor, it was possible to correct the errors, thus rendering the effects negligible, as reported in the results section.

## 1. Introduction

The purpose of the study reported here was to contribute to the development of local positioning systems (LPS) for indoor localization of mobile agents. Our research group is currently developing an LPS based on the transmission and reception of infrared (IR) signals using various strategies: Time difference of Arrival (TDoA), Time of Arrival (ToA) and Angle of Arrival (AoA). TDoA is obtained by differential phase shift between the receivers, ToA is obtained calculating the time of arrival (need synchronism between receiver and transmitter) and AoA of the measure the angle of arrival. Subsequently the position is obtained by trilateration, (TDoA and ToA cases) or triangulation (AoA case).

Studies [1,2,3] describe examples of LPS that calculate the position of a mobile robot on the basis of (TDoA) estimated from signal phase differences, using a phase-locked loop (PLL) to synchronise signals and then I/Q demodulators to calculate the phase shift. Thus, in [1], a sinusoidal signal at 6 MHz was emitted from a mobile robot and received by five photodiodes distributed in the environment. The mobile robot’s position in the environment was then calculated from the known position of these sensors. In [2], a new measurement system was proposed aimed at mitigating the effect of multipath, which generates errors in position calculations. In this case, instead of using sinusoidal signals as in [1], the agent transmitted pseudorandom codes modulated by a sinusoidal signal with a frequency greater than 25 MHz. The measurement system was based on a delay-locked loop (DLL) as the first stage of delay measurement, followed by a PLL and an I/Q demodulator. The DLL stage was used to mitigate multipath errors, improving positioning accuracy. A system for differential phase measurement was proposed in [3], but in this case the transmitters were distributed in the environment and the detector was located on the agent. To discriminate between the transmitters, these used different frequencies, and the agent’s position was obtained by calculating the phase shift between signals. As in [3], the system described in [4,5] comprised transmitters on the ceiling of the environment and a receiver located on the mobile robot. In this case, the received signal strength (RSS) from each transmitter was transformed into a distance, and the position was deduced by triangulation. In [6], the robot’s position was identified by means of cameras incorporating IRED, rendering the system robust even under poor lighting conditions. Thus, the known position of the cameras and synchronously captured images were used to calculate the position of the mobile robot. The study reported in [7] was based on the use of ultrasound. Transmitters were located on the ceiling and each mobile robot was equipped with a receiver. In this case, the transmitters emitted different Kasami codes which were separated in the receivers using the code division multiple access (CDMA) technique. Once the codes had been separated, the time difference of arrival was calculated for each, and the position was thus calculated from the known position of each transmitter.

In a novel LPS sensor system being developed by the GEINTRA group [8], position sensitive detectors (PDS) are used as IR signal detectors. Thus, the relative angle and distance to the signal source is obtained in each of these sensors and then used to deduce the agent’s position. This technique requires two accurate measurements: the depth measurement (an aspect not discussed in this paper) and the angle of arrival of the signal in a PSD equipped for this purpose with a optical system. As presented in Section 2, the currents obtained from different PSD sensor electrodes (suitably amplified) can be used to deduce the *X*-*Y* position of the point of incidence of a light beam on the sensor surface. This bright spot on the surface is the image that the optical system generates of the IR signal source based on the rays that reach it. Thus, the precise position of the point of incidence and the focal length of the optical system can be used to determine the angle of arrival.

However, it is necessary to overcome two problems in order to achieve this goal. First, the PSD sensor and the optical system are not ideal and neither is the connection between them. Consequently, it is not possible to precisely determine the intrinsic system parameters necessary for geometrical measurement (e.g., the nonlinearities of the sensor surface, the actual geometric centre of the sensor, the focal length of the optical system, the centre of the optical system in the sensor (intersection of the optical axis), nor the radial and tangential distortions of the optical system, *etc.*). Therefore, as with the cameras, it is necessary to develop a continuous geometric calibration method for the sensor (although in the case of the cameras, this a discrete system subdivided into pixels). Attempts to design a calibration method and model are further hindered by a second problem: it is not possible to obtain good calibration or deduce the actual point of incidence. This is because the position of the point of incidence (on a continuous surface) is obtained from measurements of currents by different electrodes. As a result, variations in the resistance and capacitance of different current paths due to PSD sensor defects, and above all variations and differences in the current amplification stages of the different electrodes (amplifier channels) due to component tolerances and drifts (the most important adverse effect), lead to unacceptable errors in light beam incidence detection that are then propagated to other measurement steps, giving rise to very high errors. Other aspects such as the signal frequency used, noise, suboptimal operational amplifier parameters, ADC quantization, *etc.*, also exert an effect; however, as will be shown in this article, they do so to a lesser extent.

Before addressing the development and implementation of a geometric calibration model to accurately obtain the intrinsic system parameters, it is first necessary to calibrate the electronic performance of the PSD and the different amplifier channels in order to obtain the gain balances and asymmetries and frequency-related performance of the PSD-amplifier circuit system in each channel. This latter is the subject of the study presented here.

Given the problem to be solved, the first step was to perform a search of the literature on PSD sensors, and more specifically, on their calibration. The study reported in [9] attempted to solve the problem of PSD sensor distortion by using a Kalman filter to minimise the error caused by system noise, and after the filtering step, applying a correction to the PSD sensor distortion. To this end, a correction parameter was added for the direction “*x*” and another for the direction “*y*” that depended on the point of incidence at the centre of the PSD sensor. These parameters depended on two constants calculated by mapping the PSD sensor surface with a laser. The studies reported in [10,11,12,13] used two or more PSD sensors equipped with a lens to calculate the angle of arrival of a beam of light and thus estimate the position of a mobile robot based on detection by two or more sensors using trilateration. Stereo measurement was used to calculate the position of an IRED. The position errors obtained were attributed to errors in LED emission, sensor assembly (PSD sensor + lens), low SNR, PSD sensor vibration, temperature, spot size, and PSD and lens distortion. In [14], an LPS was proposed based on a PSD sensor located on the ceiling of a room and a mobile robot equipped with an IR transmitter. This study was aimed at compensating the dynamic errors obtained using a Kalman filter to estimate position. However, the paper also reported that the largest errors were caused by PSD sensor distortion, thus highlighting the need not only to correct PSD sensor distortion but also for calibration to reduce the static error generated by each of the sensor system components.

In [15], several PSD were used to control a robot arm, mapping the sensor with a laser. This required a system in which vibrations did not affect measurements and where the entire PSD sensor surface was very accurately mapped by moving the laser in 1 µm steps. To achieve this, the PSD sensor and the laser were positioned completely parallel and the laser moved at right angles to the PSD sensor. The mapping was used to relate points on the PSD sensor to points of the lasers attached to the robot arm, eliminating errors due to PSD sensor distortion and others produced by the electrical circuit. In [16], a method was developed using PSD sensors and lasers to measure error in the rotational movement of a rotary machine. A laser diode was attached to the rotary machine, and the PSD sensor was attached to a fixed point in alignment with the laser diode when the machine rotated. The laser sweep the PSD sensor following a circular pattern, or an ellipse when the PSD sensor was not completely perpendicular to the laser. When the movement of the rotary machine was stable, the circle drawn was very similar at each rotation; however, if the machine vibrated, this produced a difference in the circles.

In the studies cited on this subject, calibration was performed to correct distortion. In all cases, it was performed by projecting a laser beam onto the sensor surface and sweeping tens and even hundreds of thousands of points. Highly complex and expensive implementation methods and infrastructures were required for this, precluding generalisation of such systems to a large scale. Moreover, this correction is only useful in cases where the system does not subsequently incorporate a lens, since this can also cause distortion. Consequently, distortion is not calibrated in our method until the second step in conjunction with the lens distortion (not described here), using geometric calibration techniques once the optical system has been attached, for which it’s necessary to correct previously the electrical errors.

Another aspect to note after reviewing the state of the art is the scant and superficial attention paid to problems generated by the amplifier circuits, signal frequency, digitization or system SNR when correcting localization errors regarding the point of incidence of the light beam (which, as demonstrated here, is necessary). Furthermore, in [12] the final localization errors obtained for a light source have been attributed to the causes described and tackled here (sensor assembly, low SNR, PSD sensor vibration, temperature, spot size, distortions, *etc.*), but without attempting to propose or perform any alternative method to mitigate them. In [9], an optical system has been attached to the sensor, using the detection results to roughly correct the position estimation obtained by the Kalman filter. Obviously, better estimates are obtained this way, but the results include the system’s measurement errors since it has not been calibrated.

Therefore, given the comments and conclusions of these studies indicating the need to analyze the influence of multiple factors, calibrate in accordance and thus mitigate the most important of these, it is evident that the static error generated by each of the sensor system components must be reduced, rendering it necessary to analyze all the causes and propose solutions to eliminate the errors generated.

This paper presents a detailed analysis of the influence on measurement of the various factors discussed in the literature and the conclusions of other previous studies. In addition, we simulated real situations (including some that were worse than would be encountered in reality) in order to obtain empirical results and present an electrical calibration method for PSD sensors. This calibration proposal is both simple and inexpensive, and also corrects imbalances between PSD sensor output signals once amplified and digitized. Furthermore, the method takes into account variations in the behaviour of the PSD sensor output signals at different frequencies, and does not require a high resolution measurement system nor a very sophisticated infrastructure.

The remainder of this paper is organised as follows: Section 2 reviews the mathematical modelling and performance of a PSD sensor. In Section 3, the errors derived from the point of incidence of a beam on the PSD sensor and the influence of its associated circuitry are analyzed and modelled. After modelling PSD performance and sources of error, Section 4 describes the method developed to calibrate variations in performance during the different stages of signal amplification. Then, the tests conducted and results obtained are presented in Section 5. The paper ends with the conclusions and suggestions for future research in Section 6.

## 2. Background: Measurement Model Formulation

**Figure 1:**
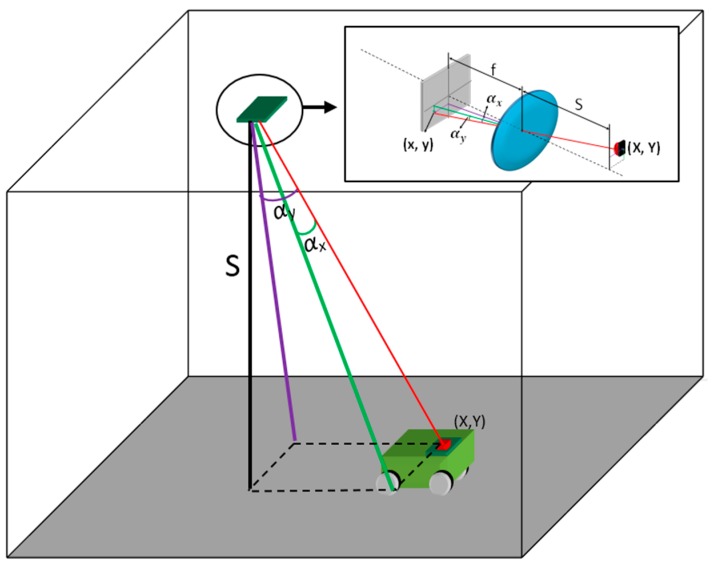
LPS System with a PSD sensor where *f* is the focal distance, *S* is the distance between emitter IR and Lent, $\alpha_{x}$ *y* $\alpha_{y}$ the angles of incidence for each axis, *x*, *y* the impact point over the PSD sensor and *X*, *Y* the point in the environment.

This LPS proposal is based on the localization of mobile agents using infrared light. A schema of this is shown in Figure 1. The basic structure consists of a PSD sensor located on the ceiling of a room, and an IR transmitter attached to the mobile agent. We know the distance between the floor and ceiling (S) so we determine the position of the mobile agent by (AoA) of the signal using a single PSD sensor. In the future, we plan to measure distance using (TDoA).

As already mentioned, LPS measurement is performed in two stages, first measuring the angle of arrival and second measuring the distance. For the first stage, it was necessary to develop a method for accurately measuring the angle of arrival of the light beam on the PSD sensor surface. This method consisted of obtaining the intrinsic system parameters necessary for angle measurement, such as focal length, optical centre and radial and tangential distortions. The intrinsic parameters were calculated from a series of measurements made with the PSD sensor. Consequently, these calculated points determine the accuracy of the intrinsic parameters and therefore the error in measuring the angle of arrival. Thus, our first goal was to obtain the point of incidence on the surface of the PSD sensor with minimal error. The following sections analyze the effects that generate measurement errors.

**Figure 2:**
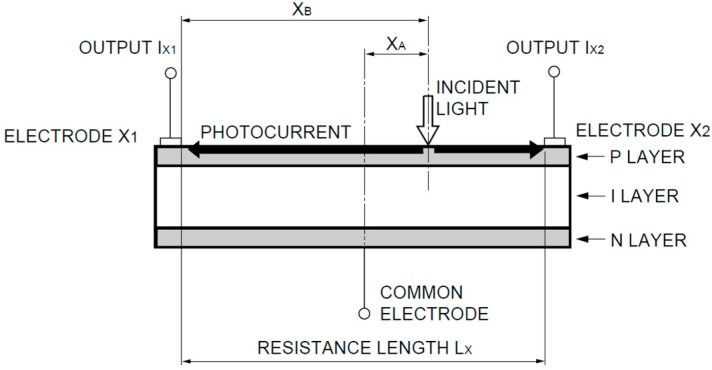
Section of a one-dimensional PSD (image courtesy of Hamamatsu, obtained from the PSD technical information).

The PSD sensor employed was a photodiode which generates a current dependent on the optical power striking its active area and on the wavelength. The PSD sensor could be equipped with either two or four anodes, and one cathode. The current flowing through each of the anodes (distribution of the total current flowing through the c athode) can be used to calculate the point of incidence of the light beam on the PSD sensor surface. Figure 2 shows a section of a one-dimensional PSD consisting of a uniform P-type resistive layer, on which the electrodes are located, over a high resistivity intrinsic layer and finally an N-type layer with a common electrode.

The photocurrent is generated in the resistive layer and is distributed between the electrodes in the ratio of electrical resistance between the electrode and the point of incidence of the light beam (which generates the current), which is proportional to the distance of the point of incidence from the electrode.

The ideal equation to calculate the point of incidence (*X_A_*) in relation to the currents flowing in the different electrodes (*I_X_*_1_, *I_X_*_2_, *I_X_*_3_, *I_X_*_4_) and the size of the one-dimensional PSD (*L_X_*) is as follows:

(1) $$X_{A}=\left(\frac{I_{X2}-I_{X1}}{I_{X1}+I_{X2}}\right)\frac{L_{X}}{2}$$

There are several types of PSD sensor, but they are all either one-dimensional and two-dimensional. The difference is that one-dimensional sensors measure the point of incidence in one direction whereas the two-dimensional ones measure it in two directions. Two-dimensional sensors can be further subdivided into duo-lateral, tetra-lateral and pin-cushion PSD. These differ in the location of the electrodes and also in the equations used to calculate the point of incidence of the light beam.

**Figure 3:**
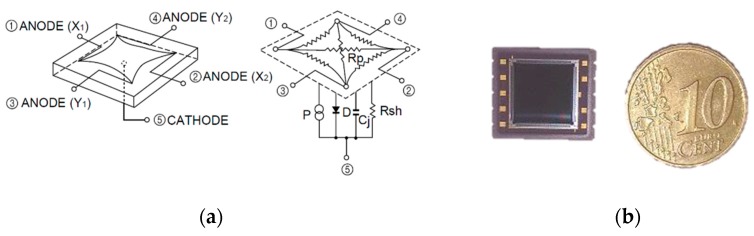
(**a**) Equivalent pin-cushion PSD circuit (image courtesy of Hamamatsu, obtained from PSD technical information); (**b**) Image of PSD S5991-01, Hamamatsu.

Pin-cushion PSD sensors offer improvements over the others, such as a lower dark current, faster response and dramatic reduction in distortion. The equivalent circuit of a pin-cushion PSD sensor is shown in Figure 3a. The equations for calculating the position *x*, *y* of the point of incidence of the light beam on a PSD under ideal conditions are:

(2) $$x=\frac{L_{X}}{2}\left(\frac{\left(I_{X2}+I_{Y1}\right)-\left(I_{X1}+I_{Y2}\right)}{I_{X1}+I_{X2}+I_{Y1}+I_{Y2}}\right)$$

(3) $$y=\frac{L_{Y}}{2}\left(\frac{\left(I_{X2}+I_{Y2}\right)-\left(I_{X1}+I_{Y1}\right)}{I_{X1}+I_{X2}+I_{Y1}+I_{Y2}}\right)$$

where *L_X_*, *L_Y_* are the sensor dimensions, and *I_X_*_1_, *I_X_*_2_, *I_Y_*_1_, *I_Y_*_2_ are the currents obtained in the PSD according to the incidence of light.

As will be discussed in the following sections, we amplify and digitize the signals to calculate the point of incidence, because is just more convenient for accuracy and simplicity. The PSD sensor must be polarised so that when the light strikes its surface, it generates a current. The currents thus generated are so small (in the order of pA) that they must be amplified in order to work with them. For this study, we used the PSD sensor model S5991-01. All the findings reported in this paper derived from its use are generalizable to other PSD pin-cushion type with the same equivalent electrical circuit, due to the imbalance of the PSD sensor pin-cushion are very small compared to the external electrical circuit imbalances. Moreover all sources of error that may have each channel will be calibrated with the sources of error due to external electrical circuit. 

**Figure 4:**
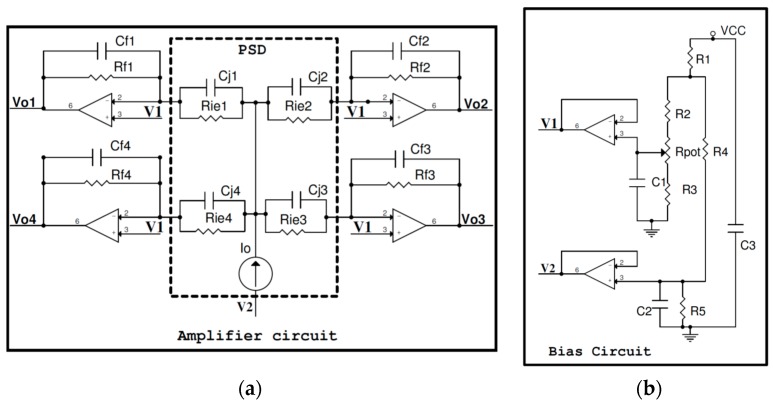
Schematic diagram of the electronic system to handle the PSD sensor (**a**) Amplifier circuit; (**b**) Bias circuit.

The current amplification and bias circuits designed for this sensor are shown in Figure 4a,b, respectively. As can be seen, the circuit makes it possible to work with output voltages (Vo1, Vo2, Vo3 and Vo4) instead of currents.

The sensor has to be polarized with reverse bias to get a current linearity proportional to the optical power received from the source of light. Since the dark current and the equivalent capacitance of the PSD depend on the bias voltage, selection of this latter will be a compromise between these factors. The bias circuit affects the amplification stage of each of the four PSD current outputs equally. Therefore, we assume that it does not affect calculation of the coordinates (*x*, *y*) because it is common to all the PSD sensor output signals, constantly maintaining the relationship between Voi signals, *i* = {1, 2, 3, 4}.

In the case of the signal conditioning circuits, we used transimpedance amplifiers to amplify the signals and perform current-voltage conversion. In addition, a capacitor was added to filter out noise at frequencies above 16 kHz (system cutoff frequency). Therefore, the ideal equations for calculating the point of incidence of the light beam on the PSD sensor surface given by the manufacturer, Equations (2) and (3), which depend on the value of the PSD sensor output currents, must be modified into expressions Equations (4) and (5), thus depend on the amplifier output voltages. These expressions remain ideal:

(4) $$x=\frac{L_{X}}{2}\left(\frac{\left(V_{o2}+V_{o3}\right)-\left(V_{o1}+V_{o4}\right)}{V_{o1}+V_{o2}+V_{o3}+V_{o4}}\right)$$

(5) $$y=\frac{L_{Y}}{2}\left(\frac{\left(V_{o1}+V_{o2}\right)-\left(V_{o3}+V_{o4}\right)}{V_{o1}+V_{o2}+V_{o3}+V_{o4}}\right)$$

In Equations (4) and (5), we have assumed that the electrical circuit components are ideal and present the same performance. However, due to the manufacturing process and sensitivity to environmental factors, in reality their magnitudes will be different to the nominal magnitude, and therefore the responses in each channel will also differ. Consequently, they will affect calculation of the point of incidence of the light beam on the PSD sensor surface. Other factors that will also affect calculation of the point of incidence include component noise (Shot noise Thermal noise and amplifiers noises), ADC quantization noise, *etc*.

## 3. Errors in Calculation of the Point of Incidence on the PSD Sensor Surface

We must know the relevance of each error to obtain the impact point in the PSD surface to determinate the position of a mobile unit in an indoor space. To simplify the analysis, an independent assessment of each one will be done. Nevertheless, a total calculation based on a model including all noises will be done in last step, in order to evaluate the influence of all of them into the final result. 

The following flow will be applied to obtain it:

- The first step will be the analysis of component tolerances influences (feedback resistance and capacitor of amplifier stage) in the variance of gains. Thus, each of four output PSD signals will be amplified by a *k_i_* factor. This amplification will produce a deviation in determining the actual impact point. Both tolerances will be modelled on an uniform distribution *U*[*a, b*], using realistic values for components and their tolerances. In addition, *k_i_* will be depended on the frequency. Although, the used frequency for this PSD is lower than 20 kHz, frequency and tolerances will be analysed together. These factors will provide a relevant error in the total analysed error, even using low tolerance values (1% for resistors and 5% for capacitors) for a working frequency of 10 kHz. The expected error under these conditions will mean a 3% of the PSD sensor size ( the error will mean 3% of the space size covered by the FOV).
- The next step to analyse will be temperature changes in amplification component values. In simulation and test stage, different profiles of working temperatures and temperature component coefficients based on realistic values will be applied. A uniform distribution *U*[*a,b*] will be modelled to include previous features. Changes in the temperature produce variations in the nominal values of components. Nevertheless, it can be considered an independent effect of tolerance components. For this situation, the estimation is that the produced error will not a notorious contribution because the temperature effect will have the same influence into all system components.
- After tolerance of components and temperature changes, the next issue to analyse will be the influence of system noise. There will be a correlation among them. The different types of noise to evaluate will be: shot, thermic and operational amplifier noise. All of them will be analysed under a white Gaussian noise (*N*(*0, σ2*)) hypothesis.
  ○ Shot noise depends on the dark current and sensor (photodiode) current. This noise source will cause the main contribution in the total error of this stage. Although, the used PSD sensor in this work (pin-cushion) produces less dark current than others, shot noise will mainly be the most remarkable error.
  ○ Thermal noise depends on the component values, changes of temperature and equivalent bandwidth. This type of noise is correlated with previous analysis (tolerance of components and temperature changes). The level of correlation between them depends on the tolerance and temperature changes but the contribution of shot noise (independent of them) has more influence that this one. To simplify the analysis, a separated evaluation of each one will be done. The influence of this error in the impact sensor point will depend on the SNR. We expect that this one will be at least 10 times lower than tolerance of components because the use of digital filters will improve SNR.
  ○ Amplifier noise is not depending of external parameters. It will only depend on the type of operational amplifier. Due to this matter, we propose to choose a low noise FET type. The use of bandpass filters to reduce signal noise eliminates the offset, and thus bias currents and offset voltage and currents at the amplifier input do not affect the system. That is the reason to not include them. In any case, Section 3.4. will take into consideration this effects.
- Other noise sources to analyse are those from the analog-digital converter. Quantification errors come from the number of bits in the analog-digital converter. As we will justify in the next sections, this type can be reduced when the number of bits is increased, SPAN will be optimized or increasing sample frequency.

As a conclusion of this flow, we have established a method with an independent analysis of error source in order to evaluate the individual contribution of each one. A complete error analysis will be presented in Section 3.5.

### 3.1. Effect of Circuit Component Tolerances

From the time of exiting the PSD sensor until reaching the acquisition stage, the signal is subject to a number of variations and disturbances such as various kinds of noise, amplification processes, *etc.* One of the main problems stems from component drift and tolerances, together with environmental conditions that affect their values; these cause variations in the gain factor of the amplifiers and the frequency behaviour of the different channels, significantly affecting the accuracy of the point of incidence.

Futhermore, variations produced by *C_j_* and *R_ie_* of the PSD sensor can be assumed, because the values of *C_j_* and *R_ie_*, are stable in whole surface of PSD sensor, however others specifics errors of each channel (as the impedance of the contacts) are include with the errors of the electrical circuit and will be calibrate together. 

Consequently, for a given current output value *I_oi_* from the different electrodes, the voltage *V_oi_* (Figure 4) obtained from the channels will not be equal, due to gain imbalances. Hence, the relationship between current and voltage for each channel can be expressed as shown in Equation (6), where *K_oi_* is the gain factor affecting each channel, which depends on the channel’s component values and the PSD response for that channel:

(6) $$I_{oi}=\frac{V_{oi}}{k_{oi}}$$

Thus, Equation (6) can be applied to the point of incidence calculation Equations (4) and (5) as shown in Equations (7) and (8), which now reflect amplifier gain factors:

(7) $$x=\frac{L_{X}}{2}\left(\frac{\xi_{1}-\xi_{2}}{\xi_{1}+\xi_{2}}\right)$$

(8) $$y=\frac{L_{Y}}{2}\left(\frac{\xi_{3}-\xi_{4}}{\xi_{3}+\xi_{4}}\right)$$

where:

$$\xi_{1}=\left(\frac{V_{o2}}{k_{o2}}+\frac{V_{o3}}{k_{o3}}\right);\;\xi_{2}=\left(\frac{V_{o1}}{k_{o1}}+\frac{V_{o4}}{k_{o4}}\right);\;\xi_{3}=\left(\frac{V_{o1}}{k_{o1}}+\frac{V_{o2}}{k_{o2}}\right);\;\xi_{4}=\left(\frac{V_{o3}}{k_{o3}}+\frac{V_{o4}}{k_{o4}}\right)$$

Equation (9) can be used to analyze how the gain factor influences calculation of the point of incidence, which represents the generic gain factor k_o_ for each channel according to the real value of its components:

(9) $$k_{o}=\frac{R_{f}}{R_{f}C_{f}2\pi f+1}$$

where *C_f_* and *R_f_* are the feedback capacitor and resistor, respectively, and *f* is the operating frequency.

Using partial derivatives with respect to *C_f_* and *R_f_*, we can calculate the increase in the gain factor ($∆k_{o}$) in relation to the increase in the capacitor and resistor values due to component tolerances, as shown in Equation (10):

(10) $$\Delta k_{o}=\left|\frac{\partial k_{o}}{\partial R_{f}}\right|\Delta R_{f}+\left|\frac{\partial k_{o}}{\partial C_{f}}\right|\Delta C_{f}=\left|\frac{1}{{\left(R_{f}C_{f}2\pi f+1\right)}^{2}}\right|\Delta R_{f}+\left|\frac{-R_{f}^{2}2\pi f}{{\left(R_{f}C_{f}2\pi f+1\right)}^{2}}\right|\Delta C_{f}$$

Another interesting factor is gain factor variation as a function of frequency. Using the first derivative of Equation (10) with respect to frequency, it is possible to calculate the frequency value at which the greatest variation occurs, as well as the maximum variation in the gain factor:

(11) $$\frac{\partial \Delta k_{o}}{\partial f}=\frac{2\pi R_{f}\left(R_{f}\Delta C_{f}-2\pi R_{f}^{2}C_{f}\Delta C_{f}f-2C_{f}\Delta R_{f}\right)}{{\left(R_{f}C_{f}2\pi f+1\right)}^{3}}$$

The frequency value which generates the maximum gain factor increase is:

(12) $$f=\frac{R_{f}\Delta C_{f}-2C_{f}\Delta R_{f}}{2\pi R_{f}^{2}C_{f}\Delta C_{f}}\text{ for }f\geq 0$$

**Figure 5:**
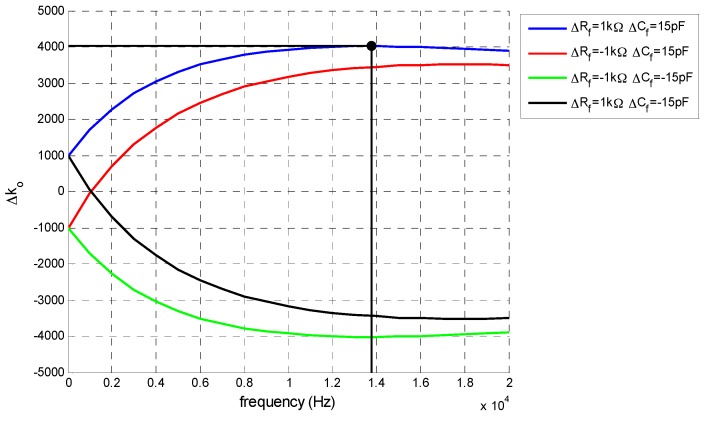
Calculation of the maximum error in the gain factor due to resistor and capacitor tolerances, in relation to the working frequency.

To illustrate this effect, Figure 5 shows the behaviour of gain factor in relation to the frequency, with a value of 100 kΩ for *R_f_* and 100 pf for *C_f_*. These values were selected considering the IR transmitter and the range of working distances, so that the output signals would be in the range of the selected ADC *SPAN*. The tolerance values selected were 1% and 15% for the resistor and capacitor, respectively. These tolerance values are common and produce a maximum difference in resistor values of ±1 kΩ and in capacitor values of ±15 pF. The simulation was performed over a frequency range between 0 and 20 kHz, because the system cutoff frequency is near to 16 kHz.

The maximum errors were extracted from the data shown in Figure 5. As can be seen in Table 1, the frequency at which maximum error occurred was 13.793 kHz. Using this frequency value in Equation (10), the maximum gain factor error obtained is 4017.9.

**Figure 6:**
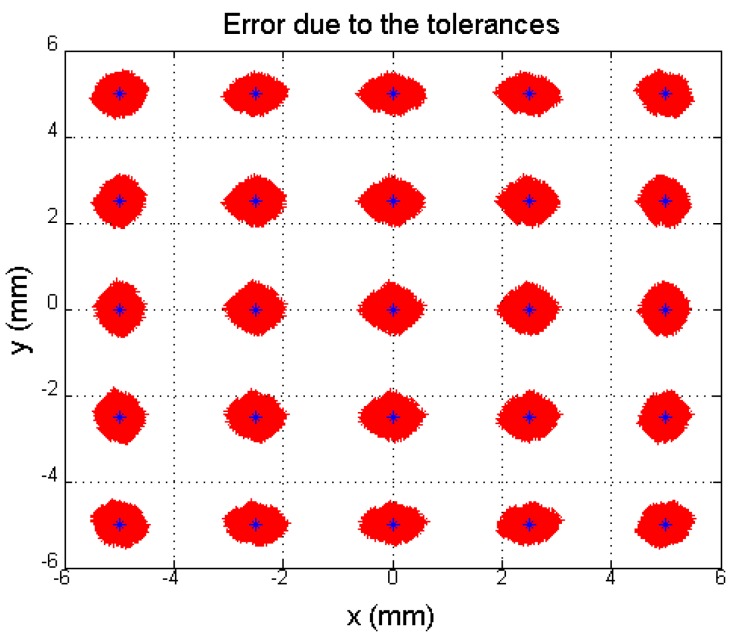
Simulation of the error in the point of incidence calculation due to tolerances.

To illustrate how component tolerance values influence channel amplification, Figure 6 shows the determination of different points of incidence of the light beam on the sensor, with a regular distribution. The blue dots represent the ideal position of the different points (the nominal component values) and red dots represent the erroneous position detected when the gain factor values are different. We simulated a uniformly distributed random variation bounded between $k_{o}=k_{o_{\text{nom}}}\pm {\Delta \text{k}}_{o}$, where $k_{o_{\text{nom}}}\;$= 53,572 (which is the gain value at 13.793 kHz) and $\Delta k_{o}$ is a uniform random variable *U* [*a, b*] bounded between ±4017.9. We continued to work with the values used to obtain Figure 5.

**Figure 7:**
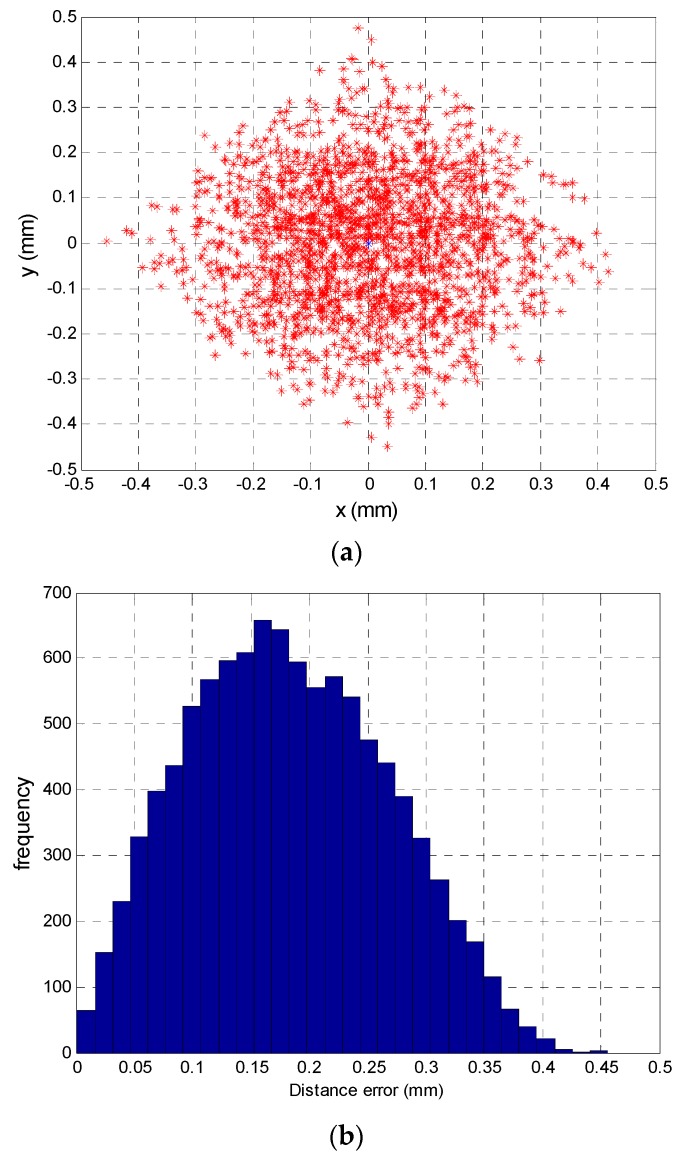
(**a**) Enlarged central point, (**b**) Distribution of distance error from the central point.

To illustrate the effect of these deviations from the ideal points in Figure 6, Figure 7a shows an enlargement of the coordinate point (0, 0) from Figure 6. Meanwhile, Figure 7b shows the distribution of the Euclidean distance error, where the maximum error is 0.4769 mm, the average error is 0.1862 mm and the standard deviation is 0.0891 mm. The error due to tolerances remains constant over the entire PSD sensor surface; however, depending on the point, the eccentricity of the error ellipse will be greater in one or another direction. Note that an error of 0.4769 mm represents 5.3% of the size of the sensor.

To illustrate the importance of this error in the development of an LPS such as that shown in Figure 1, which is aimed at detecting the position of a mobile agent using the angle of arrival at the PSD sensor of the signal emitted by an IR transmitter, note that a small error in the point of incidence calculation (E_x_ in Figure 1) results in an error in the angle of arrival (E_α_). This error also depends on the focal length (f) of the lens used, as shown in Equation (13):

(13) $$E_{\alpha }=\tan^{-1}\left(\frac{E_{x}}{f}\right)$$

For example, an error of 0.1862 mm on the PSD sensor surface (average error), using a lens with a 12 mm focal length would generate a localization error at 5 m of 77.6 mm in the position of the transmitter and 0.889 degrees in the angle of arrival related of the normal of the PSD sensor. If the focal length were 6 mm, the error at 5 m would be 155.1667 mm for position and 1.778 degrees for the angle of arrival related of the normal of the PSD sensor, in the examples we supposed that the optic system is ideal. Therefore, the angle error only is produced by the tolerances of components. The errors produced by the intrinsic parameters will be evaluated in a second phase (not include in this paper).

At this point, and after simulating the performance of the system in response to changes in component values at different signal frequencies, it can be concluded that these systematic errors constitute a very important source of error that cannot be ignored. Therefore, in addition to using high quality components, it is also necessary to calibrate their performance in order to balance the gain of the different channels. This yields the further advantage that any unbalanced systematic performance in the PSD response is also calibrated.

### 3.2. Effect of Temperature on the Electrical Components

In addition to the errors caused by tolerances and their propagation to localization of the IR transmitter, another factor that influences the nominal values of passive components is temperature. This also affects the gain factor in the amplification stage.

Variations in resistor and capacitor temperature also affect the real values, since these depend on the temperature coefficients (α*_R_* and α*_C_*). The variation in nominal component values is given in Equations (14) and (15), assuming that the capacitor shows linear variation in the range of working temperatures. Note that if the different amplifier channel and PSD components show the same values and performance with respect to temperature, there is no relative variation between the different branches, because they will all be exposed to the same temperature variations if they are located near the printed circuit board:

(14) $$\Delta R_{f}=R_{f}\alpha_{R}\Delta T^{\circ }$$

(15) $$\Delta C_{f}=C_{f}\alpha_{c}\Delta T^{\circ }$$

**Figure 8:**
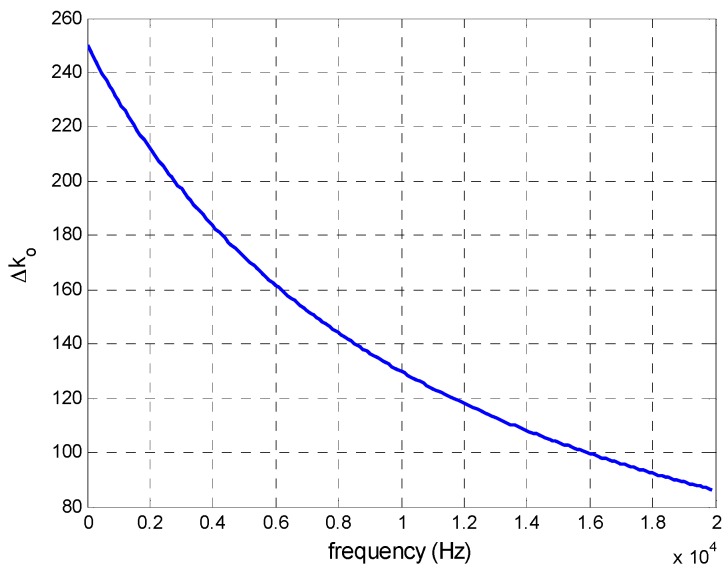
Variation in the gain factor due to a temperature difference of 5 °C with respect to signal frequency.

A simulation was performed under the following conditions: Δ*T°* = 5 °C, α*_R_* = 5 × 10^−4^ Ω/°C, α*_C_* = 0.3 × 10^−3^ pF/°C, *R_f_* = 100 kΩ and *C_f_* = 100 pF, and the result is shown in Figure 8. A value of 5 °C was selected for the differential temperature increase between the components of two channels. Since the components will be located very close to the circuit and are not exposed to different cooling, it is very unlikely that this or a greater temperature difference will occur. The temperature coefficients are given by the manufacturers, and the nominal component values were the same as those used in the case of variations caused by component tolerances (Figure 5). As can be seen in Figure 8, maximum variation in the gain factor (250) was obtained at a frequency of 0 Hz.

**Figure 9:**
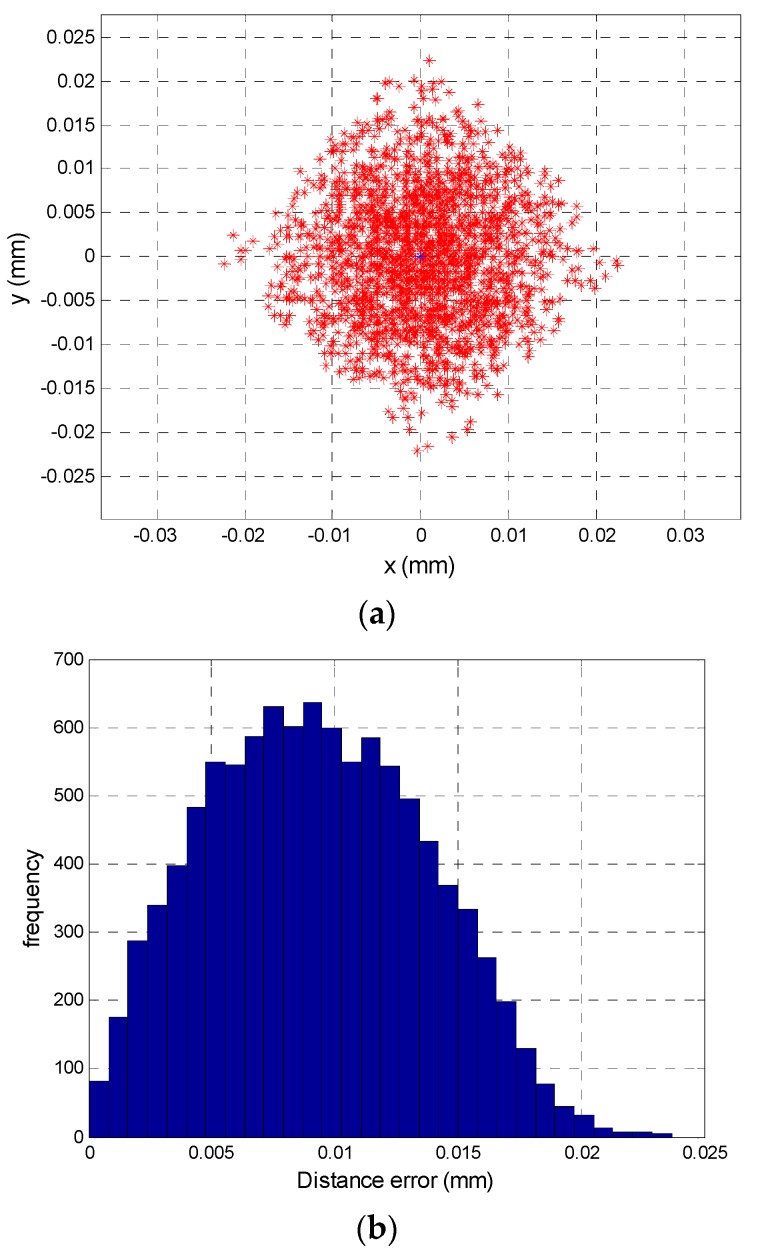
(**a**) Localisation error due to a temperature difference of ±5 °C at the central point; (**b**) Distribution of distance error for the central point.

Figure 9 depicts a simulation of localization error due to temperature variations between channels (only one point is shown), in which a uniform random variable *U*[*a, b*] has been added to the gain factor of each branch that takes values between *k_o_* = *k_o_nom_* ± Δ*k_o_*|_°C_, where *k_o_nom_ =* 1 × 10^5^ and Δ*k_o_*|_°C_ = ± 250. Figure 9a shows the error points around the coordinate point (0, 0), and Figure 9b gives the distribution of the Euclidean distance error; 0.024 mm was the largest error made in the calculation of the point on the PSD sensor surface, while the average error was 9.26 × 10^−3^ mm and the standart deviation error is 4.4218 × 10^−3^ mm and these were constant errors over the entire surface of the PSD sensor.

**Figure 10:**
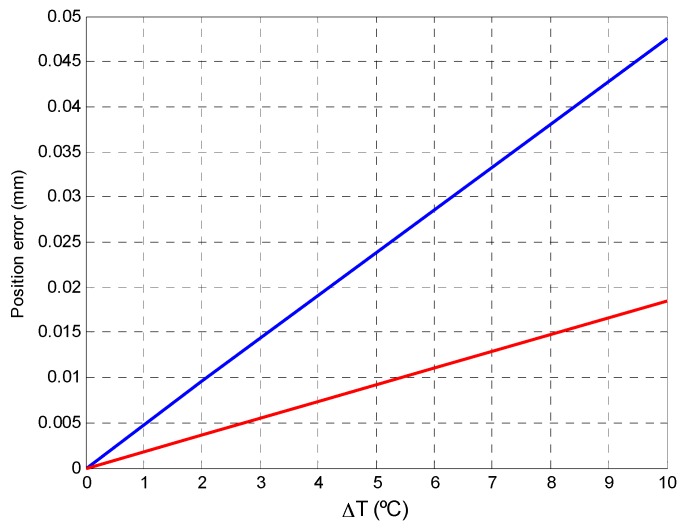
Average and maximum error in calculating the position of the point of incidence caused by large temperature differences.

Figure 10 illustrates how a large difference in temperature between the amplifiers would affect calculation of the point of incidence on the PSD sensor surface: a difference of 5 °C would cause a maximum error of 0.024 mm, an error which is 20 times lower than that calculated above for tolerances (also with maximum error values).

To conclude, we note that errors in the calculation of the point of incidence caused by temperature variations are small (an average error of 9.26 × 10^−3^ mm), especially when compared to those generated by tolerances (0.1862 mm average error). Moreover, if the design is such that relative temperature differences between the components are negligible, gain factor variations will be similar and this effect would not exist. Nevertheless, if there is a temperature difference of 2 °C between channels, the localization error for a source at 5 m would be 1.54 mm and 3.08 mm when working with focal lengths of 12 mm and 6 mm, respectively.

### 3.3. Effect of Noises on Calculation of the Point of Incidence on the PSD Sensor Surface

#### 3.3.1. Analysis of the Influence of Noises

In addition to the above mentioned problems due to gain factors at each amplification stage, a further factor which affects measurement is system noise. It is therefore necessary to analyze the SNR of the PSD sensor output and the analog-digital converter, and how this affects calculation of the point of incidence of the beam of light on the PSD surface.

**Figure 11:**
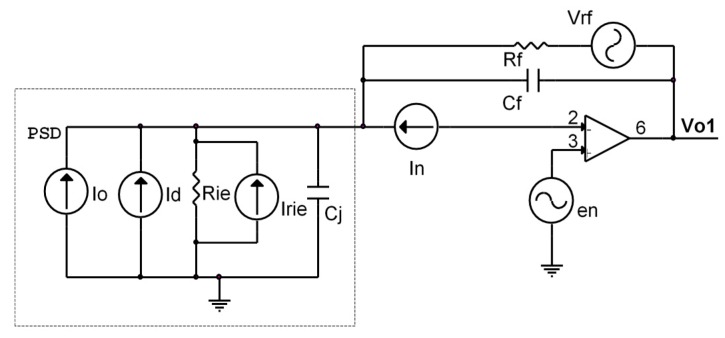
Equivalent PSD sensor circuit and electrical amplification circuit with noise.

Based on a simplified amplifier circuit consisting of a single channel (Figure 11), we analyzed the noise of the transimpedance amplifier output, *i.e.*, the noise produced by the resistors, capacitors, the PSD sensor itself and the operational amplifier.

Where *I_o_* is the photocurrent generated by the PSD sensor, *ID* represents the dark current, *R_ie_* is the interelectrode resistance, *C_j_* the junction capacitance, *R_f_* the feedback resistance, *C_f_* the feedback capacitance, en the equivalent noise input voltage of the operational amplifier, *I_n_* the equivalent noise input current of the operational amplifier and *V_n_* the total noise.

Different noise effects can be seen in the circuit in Figure 11, such as shot, Thermal or Operational. Each expression must be obtained and the effect of each one summed.

The expression for the shot noise (*V_s_*) in the circuit is:

(16) $$V_{S}=R_{f}//C_{f}\cdot \sqrt{2\cdot q\cdot \left(I_{o}+ID\right)\cdot BW}$$

where *q* is the electron charge ($1.6\times 10^{-19}$ C) and BW is the bandwidth (Hz) of the circuit.

Thermal noise (Johnson noise current) is generated by interelectrode resistance (*R_ie_*) and feedback resistance (*R_f_*), and the expressions are:

(17) $$V_{Rie}=R_{f}//C_{f}\cdot \sqrt{\frac{4KT}{R_{ie}}\cdot BW}$$

(18) $$V_{R_{f}}=R_{f}//C_{f}\cdot \sqrt{\frac{4KT}{R_{f}}\cdot BW}$$

where K is the Boltzmann constant ($1.38\times 10^{-23}$ J/K) and *T* is the absolute temperature (*K*).

The operational amplifier noises are:

(19) $$V_{en}=\left(1+\frac{R_{f}//C_{f}}{R_{Rie}//C_{j}}\right)\cdot en\cdot \sqrt{BW}$$

(20) $$V_{in}=R_{f}\cdot in\cdot \sqrt{BW}$$

The total output noise (*V_n_*) of each amplifier is:

(21) $$V_{n}=\sqrt{V_{S}^{2}+V_{Rie}^{2}+V_{R_{f}}^{2}+V_{en}+V_{in}}$$

**Figure 12:**
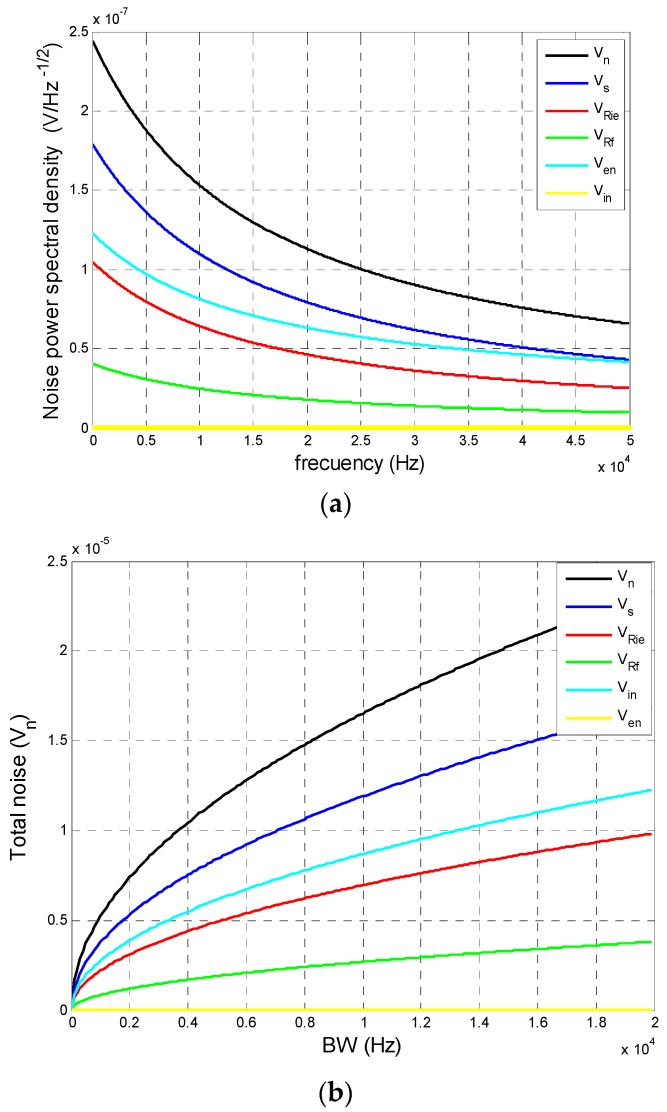
(**a**) Noise Power Spectral Densitiy of the conditioning circuit including the PSD and transimpedance amplifier; (**b**) Total noise at the amplifier output depending on the bandwidth of the bandpass filter.

Figure 12a shows the maximum noise value that can occur at the transimpedance amplifier output in relation to the frequency, obtained by simulation under the conditions indicated below. In the case of the dark current (*ID*) and photocurrent (*I_o_*), we used the most extreme (unfavourable) values given by the manufacturer for Hamamatsu PSD. Thus, the values used were: *ID* = 10 nA, *R_f_* = 100 kΩ, *C_f_* = 100 pF, *T* = 293 K, *R_ie_* = 15 kΩ, *C_j_* = 1000 pF, *en* = 16 nV/$\sqrt{\text{Hz}}$, *in* = 1.1 fA/$\sqrt{\text{Hz}}$ and *I_o_* = 0.001 mA. The results were obtained as a function of the system bandwidth (*BW*). These conditions represented a very conservative case which would be unlikely to occur in a real case. The aim of presenting this worst case scenario was show the extent to which accuracy would be affected when calculating the point of incidence on the PSD sensor surface. Figure 12b shows the total noise that would occur depending on the filtered bandwidth.

The simulation results show that system noise can be reduced by increasing the signal frequency and using filters with smaller bandwidths; however, increasing signal frequency would also reduce signal gain and the *SNR* would not improve. However, if the bandwidth is reduced, *SNR* is improved. In the case of real applications, the frequencies used are between 6 and 8 kHz, because is the center of the BW of the circuit and the attenuation at these frequencies is small.

With regard to filter, is a butterworth with BW of 1 kHz, the value of bandwidth is chosen depending on the number of coefficients, due to when more narrow is the bandwidth, the number of coefficients increased and the time of computing too. Under these conditions, total noise values are 1.796 × 10^−5^ and 1.653 × 10^−5^ V.

Having explored the evolution and impact of system noise in a worst case scenario, we analyzed how this noise affects calculation of the point of incidence on the PSD surface as a function of the SNR, and how this can be expressed. To this end, a noise factor ($\sigma_{i})$ was added to each PSD sensor signal, obtaining the expressions shown in Equations (22) and (23) for calculating the point of incidence on the PSD sensor:

(22) $$x=\frac{L_{x}}{2}\cdot \frac{\left(V_{o2}+\sigma_{2}+V_{o3}+\sigma_{3}\right)-\left(V_{o1}+\sigma_{1}+V_{o4}+\sigma_{4}\right)}{V_{o1}+\sigma_{1}+V_{o2}+\sigma_{2}+V_{o3}+\sigma_{3}+V_{o4}+\sigma_{4}}$$

(23) $$y=\frac{L_{y}}{2}\cdot \frac{\left(V_{o1}+\sigma_{1}+V_{o2}+\sigma_{2}\right)-\left(V_{o3}+\sigma_{3}+V_{o4}+\sigma_{4}\right)}{V_{o1}+\sigma_{1}+V_{o2}+\sigma_{2}+V_{o3}+\sigma_{3}+V_{o4}+\sigma_{4}}$$

The noise $\sigma_{i}$. in each of the signals is a Gaussian white noise *N(0,σ^2^)* with a standard deviation equal to *V_n_* (total noise) and the relation to SNR is determined by the expression Equation (24). Substituting in Equations (22) and (23), the coordinates for the point of incidence on the sensor surface are obtained in Equations (25) and (26), and include the relationship between SNR:

(24) $$\sigma_{i}=\frac{V_{oi}}{10^{\frac{SNR_{i}}{20}}}$$

(25) $$x=\frac{L_{x}}{2}\cdot \frac{\left(V_{o2}+\frac{V_{o2}}{10^{\frac{SNR_{2}}{20}}}+V_{o3}+\frac{V_{o3}}{10^{\frac{SNR_{3}}{20}}}\right)-\left(V_{o1}+\frac{V_{o1}}{10^{\frac{SNR_{1}}{20}}}+V_{o4}+\frac{V_{o4}}{10^{\frac{SNR_{4}}{20}}}\right)}{V_{o1}+\frac{V_{o1}}{10^{\frac{SNR_{1}}{20}}}+V_{o2}+\frac{V_{o2}}{10^{\frac{SNR_{2}}{20}}}+V_{o3}+\frac{V_{o3}}{10^{\frac{SNR_{3}}{20}}}+V_{o4}+\frac{V_{o4}}{10^{\frac{SNR_{4}}{20}}}}$$

(26) $$y=\frac{L_{y}}{2}\cdot \frac{\left(V_{o1}+\frac{V_{o1}}{10^{\frac{SNR_{1}}{20}}}+V_{o2}+\frac{V_{o2}}{10^{\frac{SNR_{2}}{20}}}\right)-\left(V_{o3}+\frac{V_{o3}}{10^{\frac{SNR_{3}}{20}}}+V_{o4}+\frac{V_{o4}}{10^{\frac{SNR_{4}}{20}}}\right)}{V_{o1}+\frac{V_{o1}}{10^{\frac{SNR_{1}}{20}}}+V_{o2}+\frac{V_{o2}}{10^{\frac{SNR_{2}}{20}}}+V_{o3}+\frac{V_{o3}}{10^{\frac{SNR_{3}}{20}}}+V_{o4}+\frac{V_{o4}}{10^{\frac{SNR_{4}}{20}}}}$$

**Figure 13:**
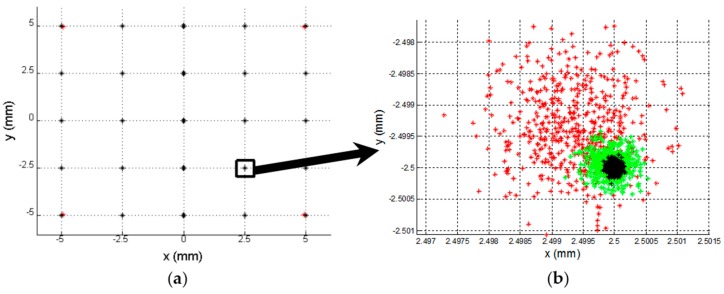
(**a**) SNR: 40 dB (red), 50 dB (green) and 60 dB (black) for 25 PSD sensor positions; (**b**) Enlarged.

To illustrate the effects of the noise quantitatively and graphically, Figure 13 shows the results of the simulation. These were obtained working with a RMS value of a sinusoidal signal at a frequency of 8 kHz, a sampling rate of 125 kHz, an acquisition time of 0.1 s and an amplitude of 1. A noise signal was added with a normal distribution corresponding to a *SNR* of between 20 and 90 dB, repeating the simulation 1000 times for each value. In Figure 13, the simulation of a *SNR* of 40 dB is shown in red, of a *SNR* of 50 dB in green and of a *SNR* of 60 dB in black. Meanwhile, Table 2 shows the average distance error, the standard deviation and the maximum error distance for the position enlarged in Figure 13. Note that the ideal points (which would be obtained with no noise) are (0, 0) with increments of 2.5 mm in each direction.

The error due to noise was low compared with those caused by tolerances and/or temperature. Note that the RMS value was used to calculate the point of incidence on the PSD sensor.

As previously, when these errors were transferred to errors in localization of the source at 5 m, a SNR of 40 dB produced an average error of 0.27 mm and 0.55 mm in the position of the transmitter with a focal length of 12 mm and 6 mm, respectively. In conclusion, if the components are suitably chosen, this error could be assumed in the measurements. However, working with higher *SNR* values would attenuate this effect (it would be 10 times lower with 60 dB).

#### 3.3.2. Analysis of the Influence of Quantization Noise (*SNR_Q_*)

In addition to system noise, there is also quantization noise. This noise depends on the ADC (number of bits, sampling frequency, *SPAN* and full scale input). The objective of this analysis was to determine which ADC parameters should be used in the conversion so that *SNR_Q_* is such that the effects of quantization are rendered negligible.

The relation between the *SNR_Q_*, the number of bits, sampling frequency and full scale can be found starting from expression Equation (27):

(27) $$SNR_{Q}=10\cdot \log_{10}\left(\frac{P_{x}}{P_{n}}\right)$$

where *P_x_* is the signal strength and *P_n_* is the quantization noise power.

*P_x_* and *P_n_* were modelled as shown in the expressions Equations (28) and (29), respectively, where Equation (28) is the signal strength for a sinusoidal amplitude signal $\frac{Vpp}{2}$:

(28) $$P_{x}=\frac{V_{PP}^{2}}{8}$$

(29) $$P_{n}=\frac{\sigma_{Q}^{2}}{fs/\left(2\cdot BW\right)}$$

where $\sigma_{Q}^{2}$ is the variance of the total quantization noise, *f_s_* is the sampling frequency and *BW* is the bandwidth of interest. $\sigma_{Q}^{2}$. is modelled as the variance of a uniform probability distribution with limits [-q /2, q /2], where q is the resolution of the ADC, which is defined by the *SPAN* between the range of values that the ADC can digitize, *i.e.*, the number of bits of the ADC. Therefore, $P_{n}$ becomes:

(30) $$P_{n}=\frac{\frac{V_{in}^{2}}{12\cdot 2^{2n}}}{fs/\left(2\cdot BW\right)}$$

Substituting *P_x_* and *P_n_* in Equation (27), we obtain the expression Equation (31), which relates ${SNR}_{Q}$ with the number of bits, full scale input and sampling frequency of the ADC, as well as the amplitude of the signal to digitize:

(31) $${SNR_{Q}|}_{dB}=10\cdot \log\left(\frac{3}{2}\right)+10\cdot \log\left(4^{n}\right)+10\cdot \log{\left(\frac{V_{PP}}{SPAN}\right)}^{2}+10\cdot \log\left(\frac{fs}{2\cdot BW}\right)$$

**Figure 14:**
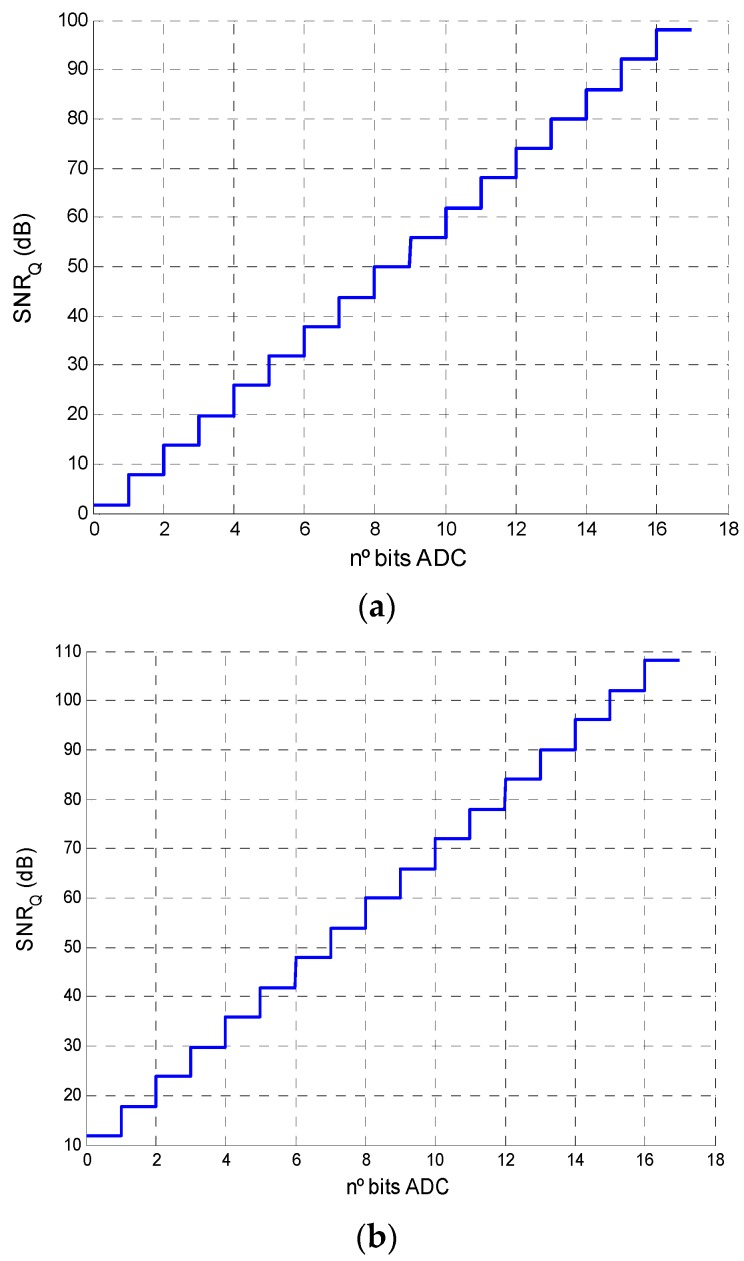
Quantization SNR in relation to the number of bits of the ADC, with Vpp = SPAN. (**a**) f_s_ = 2 × BW; (**b**) f_s_ = 20 × BW.

Figure 14a shows the value of *SNR_Q_* (dB) relative to the number of bits of the converter, with *Vpp* = *SPAN* and *f_s_* = 2·*BW*. Note that this bandwidth represents the worst case scenario. Since the working frequencies were not very high, it would be feasible to work with sampling frequencies 10 times higher or more, which would yield the results shown in Figure 14b.

From Equation (31), it is possible to calculate the ADC parameters that render quantization *SNR* sufficiently high to be negligible compared with other sources of error in the point of incidence calculation. In order to render the error generated by sampling negligible, the value for *SNR_Q_* should be 20 dB higher than the system *SNR*. In conclusion, if we assume a low *SNR* for the system (40 dB), it would be sufficient to select an ADC of *f_s_* = 120 kHz and 7 bits, for a 12 kHz signal.

### 3.4. Effect of Suboptimal Operational Amplifier Parameters

**Figure 15:**
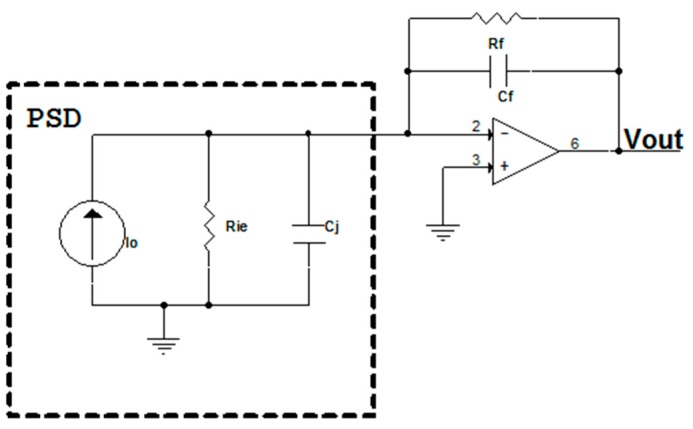
Amplifier I/V converter circuit.

Lastly, we analyzed the effects related to suboptimal performance of the operational amplifiers in a transimpedance configuration (Figure 15). The effects of a suboptimal amplifier are described in [17]. It is important to determine the existence of errors due to the effects of impedance of input offset voltage and current at the input (*V_os_*, *I_os_*), bias currents, power supply rejection ratio (*PSRR*) and slew rate.

The use of bandpass filters to reduce signal noise eliminates the offset, and thus bias currents and offset voltage and currents at the amplifier input do not affect the system. However, in the case of working with continuous signals (DC), these would have to be taken into consideration.

With regard to input impedance, it is important to select an amplifier with low input capacitance, because this to ensure system stability. This capacitance could also be compensated by adding a feedback capacitor, ensuring circuit stability, but the bandwidth will be reduced.

A limitation of suboptimal operation is the slew rate, which determines the frequency at which the operational amplifier can follow an input signal, *i.e.*, obtain the amplified input signal at the amplifier output without distortion. For a sinusoidal signal, the amplifier must have a slew rate that fulfils expression Equation (34), where *F_max_* is the maximum operating frequency and *V_P_* is the signal amplitude:

(32) $$Slew\;rate\geq f_{\max}2\pi V_{P}$$

Another aspect to consider is the *PSRR*. Because the power supply presents voltage fluctuations, the operating point of the amplifier changes and therefore the amplified signal will be affected according to Equation (35). This effect can be reduced by adding a voltage regulator between the power supply and the amplifiers, which eliminates fluctuations:

(33) $$V_{ripple\_out}=\frac{V_{ripple\_in}}{PSRR}$$

Based on the above requirements, we selected the AD824A operational amplifier (Analog Devices). This was not the only possibility: there are others with similar or better characteristics that could have been selected. The AD824A is a FET amplifier with low noise, high input impedance and a sufficiently rapid slew rate to follow signals above 31.8 kHz where *V_P_* = 10V. For the *PSRR*, a regulator such as the Cosel ZUW61215 can be added with ripple voltage ≤ 150 mV_pp_. This output ripple regulator does not affect the measurement because the ripple frequency is low, around 100 Hz, and is therefore easily filtered by the 1 kHz bandwidth digital filter, set here at a working frequency above 6 kHz.

### 3.5. Simulation with All Sources of Error

To determine the error in the position of the mobile agent with all sources of error afore mentioned, we have made a simulation based on a complete model.

The gain factor at 13 kHz, has been calculated with values for feedback resistor and capacitor of 100 kHz and 100 pF and tolerances of 1% and 15% respectively, the temperature coefficients have been 5 × 10^−4^ for resistance and 0.3 × 10^−3^ for capacitor, with a variation of 5° of temperature, being the sources of error (tolerances and temperature) uniform random variables *U*[*a, b*].

The other source of error have been the noise, being modeled as Gaussian white noise *N*(0*, σ*2) the test has been simulated with a SNR value of 50 dB and the quantization noise has been simulated with SNRQ of the 68.75 dB equivalent to a 10-bit adc, fs = 10 × BW and Vpp = SPAN. Equations (34) and (35) show the model with all sources of error:

(34) $$x=\frac{L_{x}}{2}\cdot \frac{\left(\frac{V_{o2}}{K_{o2}}+\sigma_{2}+\frac{V_{o3}}{K_{o3}}+\sigma_{3}\right)-\left(\frac{V_{o1}}{K_{o1}}+\sigma_{1}+\frac{V_{o4}}{K_{o4}}+\sigma_{4}\right)}{\frac{V_{o1}}{K_{o1}}+\sigma_{1}+\frac{V_{o2}}{K_{o2}}+\sigma_{2}+\frac{V_{o3}}{K_{o3}}+\sigma_{3}+\frac{V_{o4}}{K_{o4}}+\sigma_{4}}$$

(35) $$y=\frac{L_{y}}{2}\cdot \frac{\left(\frac{V_{o1}}{K_{o1}}+\sigma_{1}+\frac{V_{o2}}{K_{o2}}+\sigma_{2}\right)-\left(\frac{V_{o3}}{K_{o3}}+\sigma_{3}+\frac{V_{o4}}{K_{o4}}+\sigma_{4}\right)}{\frac{V_{o1}}{K_{o1}}+\sigma_{1}+\frac{V_{o2}}{K_{o2}}+\sigma_{2}+\frac{V_{o3}}{K_{o3}}+\sigma_{3}+\frac{V_{o4}}{K_{o4}}+\sigma_{4}}$$

**Figure 16:**
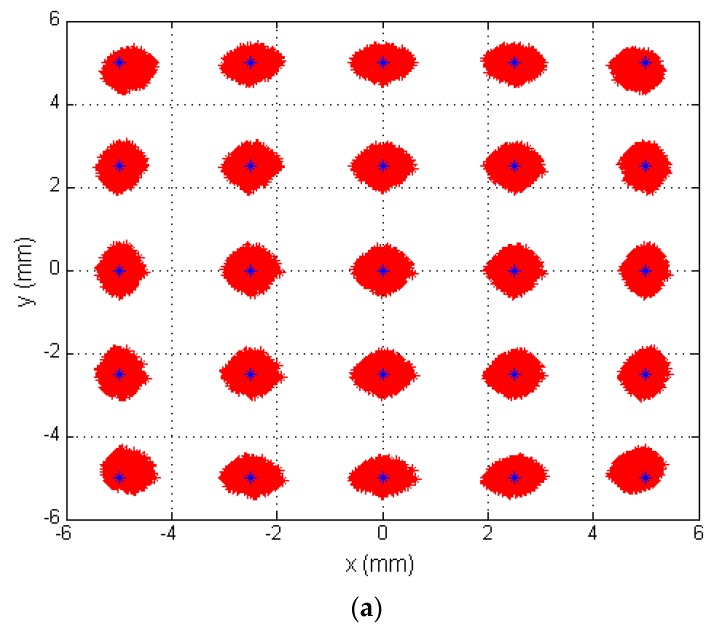

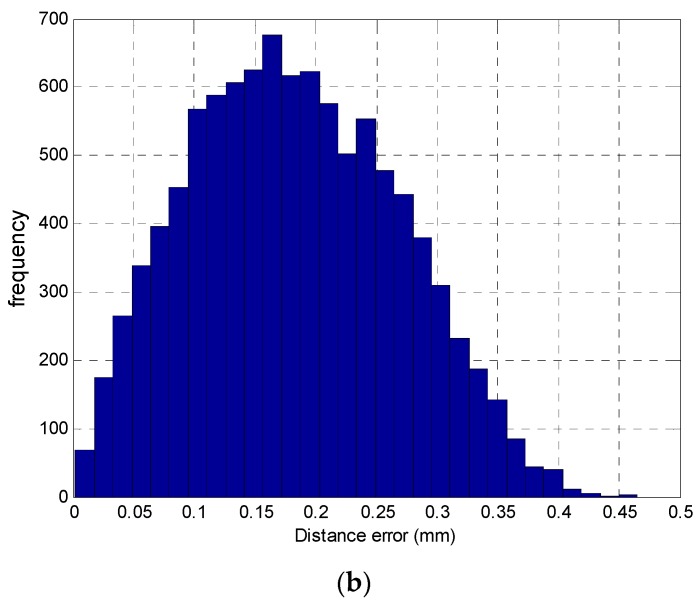
(**a**) Simulation of the error in the point of incidence calculation; (**b**) Distribution of euclidean distance error for the central point.

Figure 16a shows the determination of different points of incidence of the light beam on the sensor. The blue dots represent the ideal position of the different points (the nominal component values and without noise) and red dots represent the erroneous position detected when the gain factor values are different (due to the tolerances and temperature) and noises.

Figure 16b shows the distribution of the Euclidean distance error of the point (2.5, −2.5), being the average error is 0.2056 mm, the maximum error is 0.4617 mm and the standard deviation is 0.1177 mm, these results have been obtained by performing the test 10,000 times.

The results are similar to the error due to tolerances where the average error was 0.1862 mm for 0.2056 mm, maximum error was 0.4769 mm for 0.4617 mm , and the standard deviation was 0.0891 mm for 0.1177 mm, we can see the average error is higher, however the errors are similar. The difference of the average error in the position of agent mobile with 12 mm of focal length is 77.6 mm with just the error due to tolerance and 85.7 mm of average error with all sources of error.

### 3.6. Conclusions Drawn from the Error Analysis

From simulation results, we can check that our system has more influence to the errors from tolerances of components and temperature changes than system and quantification error. As follows, we show the system sensibility for different error source:

- The sensibility of a resistance tolerance (1%) in the impact point achieves 0.0237 mm (2.3% of complete sensor surface) for a nominal value of 100 kΩ. This error can be considered as lineal relation.
- In the case of the capacitor, its sensibility is due to its tolerance and working frequency. Thus, for a 100 pF device working in 1 kHz, the average error is 0.0021 mm. If the working frequency is increased to 8 kHz, it will produce an average error of 0.0116 mm. The average error for 16 kHz will be 0.018 mm.
- The sensibility of temperature changes has a linear relation of 0.00124 mm/°C average error to obtain the impact point.
- According to Table 2, the cases of system and quantification error depend on the SNR. The relation is a 10 times error reduction per an increase of 20 dB. In system noise case for 40 dB SNR, we achieve an average error up to 6.56 × 10^−4^ mm. This figure is decreased to 6.39 × 10^−5^ mm when SNR reaches 60 dB.

The effects analyzed in this third section (tolerances, temperature, noises, quantization noise and effects due to suboptimal OA parameters). can be divided into those that produce random errors, such as system and quantization noise, and those that produce systematic errors, such as the effect of tolerances and temperature.

In the case of systematic error, we have shown that this is a very significant source of error that cannot be disregarded and which must be eliminated. Fortunately, since it is systematic, it is possible to propose a system model and calibration method to obtain the performance parameters of the model and balance the performance of the different channels. The most important aspect and the one that requires calibration is that of tolerances. As regards the temperature coefficients, it may be sufficient to be careful with component placement in the printed circuit board design, although this is also easy to calibrate. Random errors cannot be calibrated, but as we have seen, the effect of these is negligible provided that certain criteria are observed when selecting components and their characteristics. In the following section, we propose a calibration method and a model for the gain behaviour of the different channels, which mitigate localisation error.

## 4. Calibration Method

Having identified the different sources of error associated with a PSD sensor, it is necessary to correct the variations in performance that arise during the stages of electrical circuit amplification so that they are eliminated. In other words, so that the currents are identically conditioned. We have therefore developed a calibration method that calculates variations in the gain factor between signals. This method is based on uniform illumination of the PSD sensor surface so that the current generated by the PSD is distributed equally between its terminals. Thus, the amplitude differences obtained in the amplifier outputs are due to variations in PSD performance and the gain factor of each channel.

**Figure 17:**
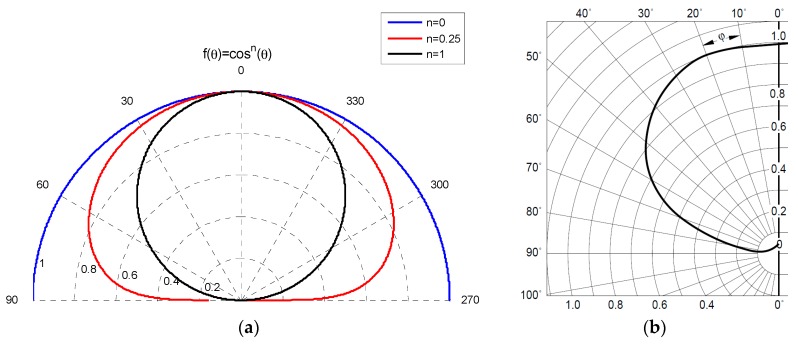
(**a**) Lambertian emission pattern with an index *n* = 1, 0.25 and 0; (**b**) Representation of the emission pattern of a commercial transmitter with n < 1.

In order to illuminate the sensor uniformly, we used an IR transmitter placed at a sufficient distance to ensure that the solid angle covering the PSD sensor was small and the emitted intensity (Ie) in each of the directions covered by the solid angle could be safely approximated to a constant value. In this respect, it is also possible to vary the IRED emission pattern. For example, in the case of a transmitter with a Lambertian emission pattern with an index *n* = 1 (Figure 17a), the difference in emitted intensity at 0° and 2° is 0.06%. If a transmitter is selected with *n* < 1, this percentage is drastically reduced. In addition, to achieve greater simplicity and accuracy without having to build more complex transmitter elements, commercial elements with n < 1 can be employed, which maintain a constant emitted intensity in a large solid angle (Figure 17b, commercial element) and ensure that the PSD sensor is illuminated homogeneously.

**Figure 18:**
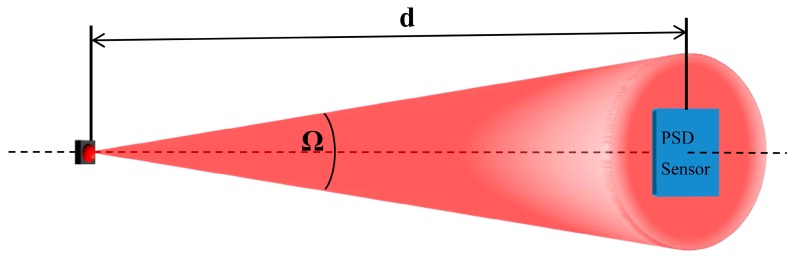
Uniform illumination of the PSD sensor.

As shown in Figure 18 the solid angle covering the PSD depends on the distance at which it is located, as well as its size. Also note that the power level received by the PSD sensor decreases with the square of the distance, and it is thus necessary to reach a compromise between the angle of arrival and distance.

The procedure for calibrating the PSD sensor was as follows:

1. To ensure that the PSD sensor was located in an area of uniform illumination, we centred the PSD sensor with the IRED transmitter, and then moved the centred sensor in all directions to verify that there were no variations in the PSD sensor output signals (Figure 18).
2. Once uniform illumination had been ensured, we measured and recorded the gain values for each channel, performing multiple tests to obtain a mean value and standard deviation. This procedure was repeated with signals at different frequencies, as the performance of the signal amplification stage depends on the frequency.

It is important to obtain the gain ratio between different channels. Therefore, one of the channels was taken as a reference and its ratio calculated in order to subsequently compensate the measurements. In this case, we used channel 1 as the reference.

3. A second degree function (*G_i_*) which is dependent on frequency (*f*) was used as a model of the correction, as shown in Equation (36):
  (36) $$G_{i}=a_{1j}\cdot f^{2}+a_{2j}\cdot f+a_{3j}$$
  where *j* = {2, 3, 4}.

This model corrects the gain factor differences in the worst cases, although a linear or constant relationship throughout the frequency range can be obtained with some of them (depending on the PSD and components used).

The parameters *a_ij_* were calculated for each of the branches by means of least squares, using the equation *A · x* = *b*. The solution that minimised the norm was:

(37) $$x={\left(A\prime \cdot A\right)}^{-1}A\prime \cdot b$$

where:

$$A=\left(\begin{array}{ccc} f_{1}^{2} & f_{1} & 1\\ \vdots & \vdots & \vdots \\ f_{n}^{2} & f_{n} & 1 \end{array}\right);\;x=\left(\begin{array}{c} a_{1j}\\ a_{2j}\\ a_{3j} \end{array}\right);\;b=\left(\begin{array}{c} {\frac{V_{ref}}{V_{j}}|}_{f_{1}}\\ \vdots \\ {\frac{V_{ref}}{V_{j}}|}_{f_{n}} \end{array}\right)$$

4. Once these parameters have been calculated, Equations (4) and (5), which are the ideal equations for calculating the point of incidence on the PSD sensor surface, become Equations (38) and (39), introducing the parameters in order to correct systematic errors at different frequencies. *V_i_* represent the values for corrected output voltage:
  (38) $$x=\frac{L_{X}}{2}\left(\frac{\left(V_{2}+V_{3}\right)-\left(V_{1}+V_{4}\right)}{V_{o1}+V_{2}+V_{3}+V_{4}}\right)$$
  (39) $$y=\frac{L_{Y}}{2}\left(\frac{\left(V_{1}+V_{2}\right)-\left(V_{3}+V_{4}\right)}{V_{1}+V_{2}+V_{3}+V_{4}}\right)$$

Thus, taking signal 1 as the reference, we obtain:

$$\begin{array}{cc} V_{1}=V_{o1} & V_{2}=V_{o2}\left(a_{11}\cdot f^{2}+a_{21}\cdot f+a_{31}\right)\\ V_{3}=V_{o3}\left(a_{12}\cdot f^{2}+a_{22}\cdot f+a_{32}\right) & V_{4}=V_{o4}\left(a_{13}\cdot f^{2}+a_{23}\cdot f+a_{33}\right) \end{array}$$

In conclusion, this is a simple calibration method for correcting systematic system errors relatively accurately, as will be shown in the results section. The method eliminates significant measurement errors and also avoids the propagation of these to the intrinsic system parameters when it is necessary to include and calibrate an optical system, as was the case in our study.

## 5. Experimental Tests

Having analyzed the problems affecting measurement error with a PSD sensor and proposed a method to calibrate systematic errors, we performed experimental tests to corroborate the results and conclusions obtained from simulations.

The tests were carried out using a PSD S5991-01 sensor (Figure 2b) and an amplification circuit similar to the one shown in Figure 4. AD824 operational amplifiers were used, with 100 kΩ resistors and 100 pF capacitors. The transimpedance amplifier outputs were connected to a PCI-6829 digitizer card (National Instruments, Austin, TX, USA). The IR transmitter used in the tests was a SFH-4233 (Osram, Munich, Germany).

**Figure 19:**
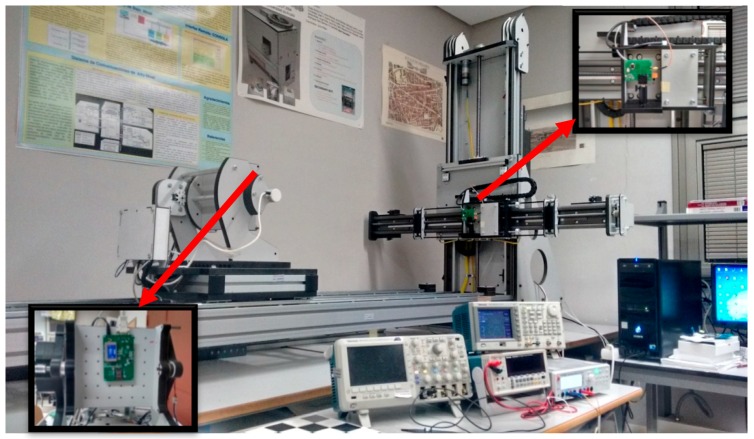
Infrastructure used for calibration.

The infrastructure employed to carry out the tests is shown in Figure 19, and had a bench with five degrees of freedom: three translation axes with a localisation error of less than 0.1 mm and two rotation axes with errors of less than 0.3°. Figure 19 also shows details of the transmitter and receiver circuits (PSD).

### 5.1. Calibration of Gain in Different Channels

The first test was to perform electrical calibration with the method and model explained in Section 4 using the transmitter indicated. To ensure that the PSD sensor was uniformly illuminated, the sensor was positioned at a distance of 1 m from the IR transmitter so that the maximum solid angle covered by the detector was 8.72 × 10^−5^ Sr. In addition, the transmitter and receiver were positioned so that, as far as possible, they were facing one another (on the same axis). Given the value of the solid angle covered, their axes did not completely coincide; nevertheless, homogeneous illumination could be assumed. In fact, sufficient margin remained to make small shifts in the relative position and confirm that the output currents were the same in different positions (uniform and constant illumination).

**Figure 20:**
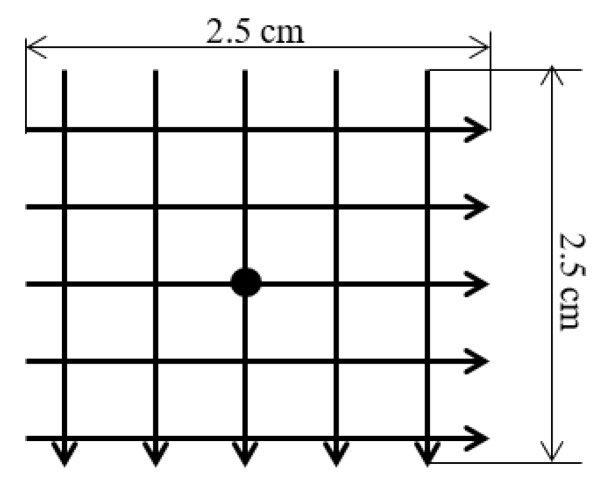
Changing the position of the PSD sensor to confirm illumination.

To ensure that the sensor was evenly illuminated, we made small changes to the position of the PSD sensor in various directions and recorded the RMS value of the signals to confirm that they did not change. Figure 20 shows the position changes implemented for this test; we performed 5 vertical scans in 5 horizontal positions. Given an initial point as a calibration point, here depicted as the centre of the grid, the total length of displacement scanning was 2.5 cm, sampling at 25 points and taking 10 samples per point.

**Figure 21:**
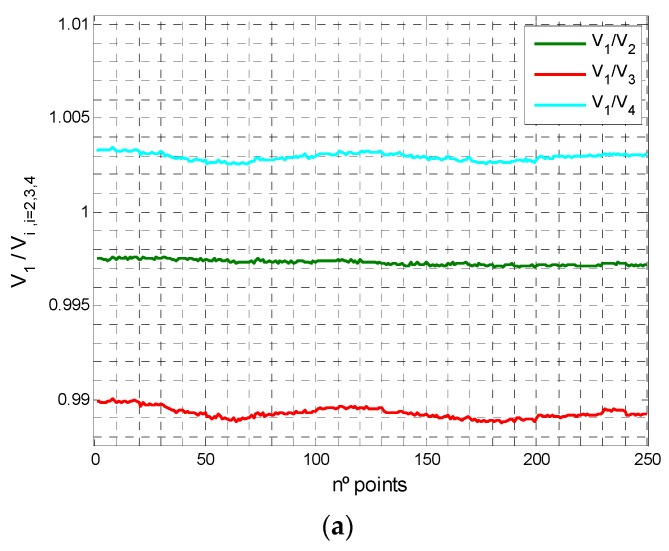

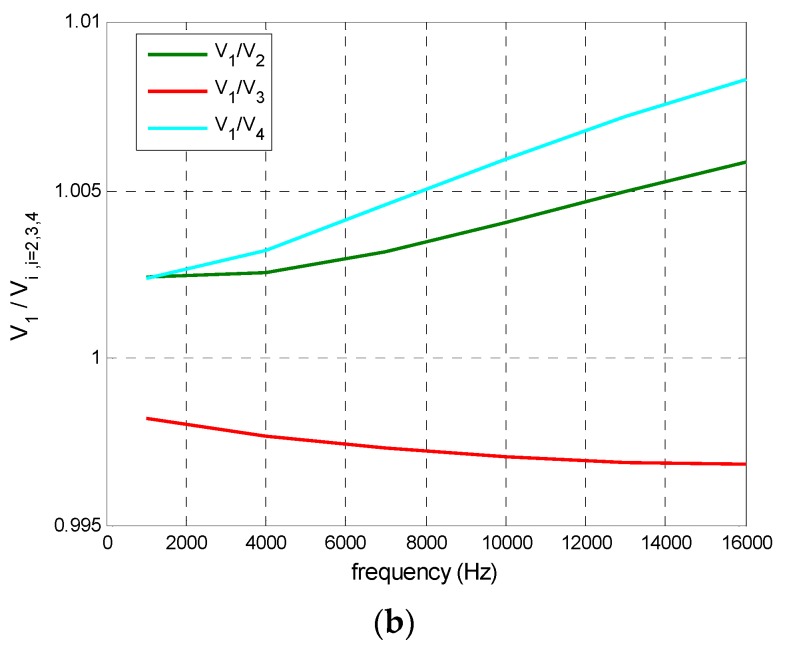
(**a**) Voltage ratios between channels; (**b**) Behaviour of these ratios with frequency.

Several tests were performed to test the effects of the PSD and the amplifiers. For one of these, we used very high quality components (resistors of 0.1% and capacitors of 2%) in order to determine the other effects unrelated to these that unbalanced the amplification channels. Figure 21a shows the relationship between signals 2, 3 and 4 with respect to signal 1 for a frequency of 8 kHz. As mentioned earlier, 250 samples were taken in 25 positions, obtaining stable values for all of these. Hence, the PSD sensor was uniformly illuminated. The standard deviations for the ratios between signal 1 and the others were 1.22 × 10^−4^ for signals 1 and 2; 1.96 × 10^−4^ for signals 1 and 3; and 1.62 × 10^−4^ for signals 1 and 4, while mean values were 0.99544, 1.00115 and 0.99372 between signal 1 and signals 2–4, respectively. Figure 21b depicts the variation in signal ratios with respect to frequency.

Since the values obtained to verify the calibration positioning were stable, those obtained in the samples 130 to 140 (averaged) were used to calibrate the gains of different channels, althought the relationship between signals are stable in the position where we have taken the samples 130 to 140 the signals were highest.

Note that in the Figure 21a shown the relationship between signals.

Thus, using the expressions proposed for this in Equation (36), we calculated the differences between signals 2–4 with respect to 1, obtaining the following results:

$$G_{2}=\frac{V_{1}}{V_{3}}=6.211\cdot 10^{-12}\cdot f^{2}+8.421\cdot 10^{-7}\cdot f+0.9882$$

$$G_{2}=\frac{V_{1}}{V_{3}}=-6.183\cdot 10^{-12}\cdot f^{2}-4.304\cdot 10^{-7}\cdot f+1.0171$$

$$G_{3}=\frac{V_{1}}{V_{4}}=-2.411\cdot 10^{-12}\cdot f^{2}+1.166\cdot 10^{-6}\cdot f+0.9875$$

These results indicate that in our case with the last parameter is enough, because our frequencies are small, but in cases with smaller PSD sensors, working frequencies could be higher and all three parameters are needed. However, it could vary depending on the PSD manufacturer, model and circuit, and therefore the proposed model was maintained. Furthermore, in the last polynomial parameter a difference was observed in the gain factors that cannot be disregarded and must be corrected.

**Figure 22:**
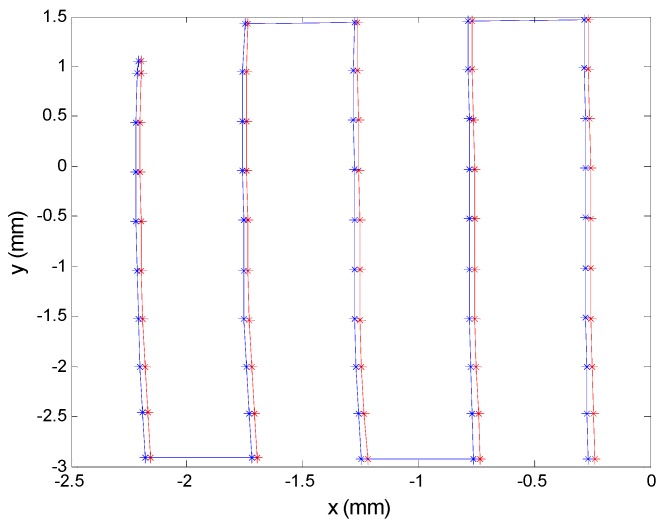
Points obtained from PSD signals using resistors with a tolerance of 0.1% and capacitors with a tolerance of 2%. Points corrected after calibration are shown in blue.

Figure 22 shows the results with the points delivered by the PSD: uncorrected points are shown in red and corrected points in blue. Note that although the two points are very close, mainly due to the high quality of the components used, there is still an error that requires calibration. The average error in the sensor was 0.029 mm, representing 0.32% of the size of the sensor. If the error were transferred to the localization of a source at 5 m distance, this would imply an error of 24.1 mm with a focal length of 6 mm, and 12 mm with a focal length of 12 mm. In conclusion, it is evident that the system requires calibration procedure. Therefore it is not mandatory to use high precision components. Actuarlly, the main advantages to use high precision components are the small deviations of nominal values from environmental conditions and/or living time of the component. Thus, this components can provide us no new calibrations because we will assume that they will not change.

While these errors may seem small and do not translate into large localization errors when the performance of the optical systems is ideal, if a sensor point localization error of 0.029 mm were transferred to the model and geometric calibration process (including the transfer function of the optical system), it would propagate and magnify errors when calculating the position of a source in 3-D space.

It can also be seen in Figure 22 that distortion became clearly evident as we moved away from the centre of the PSD toward the surface edges (*i.e.,* *x* < −2 mm). As mentioned earlier, in our case distortion will be corrected in a second geometric calibration phase, once an optical system has been attached to the sensor (not discussed here).

**Figure 23:**
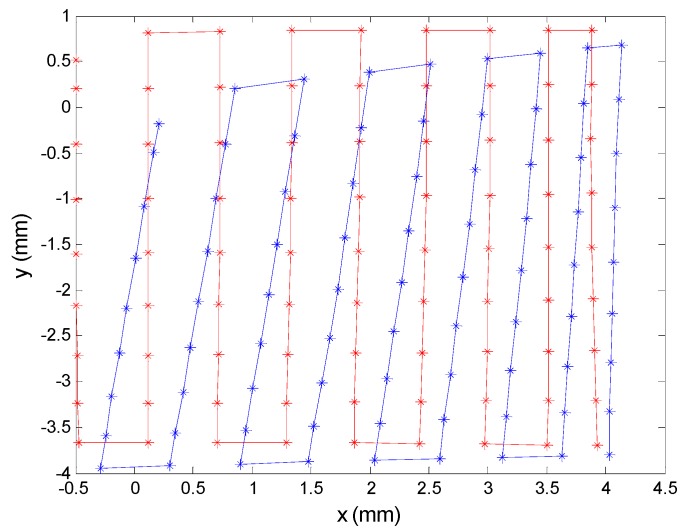
Corrected points (red), varying a resistor by 50% and points detected before correction (blue).

To illustrate the real effects of variations in component magnitudes when using higher tolerances, we varied one of the resistors by setting a value of 50%. Although this is a worst case scenario unlikely to occur in reality, we wished to demonstrate what the effects would be. As can be seen in Figure 23, the values obtained after system calibration varied dramatically with respect to those obtained prior to calibration.

Based on the simulation, these results were in the range of expected values. Note that in this case, one channel became extremely unbalanced with respect to the other three; consequently, the error near the centre of the sensor (0, 0) was very large, but decreased sharply as we moved away from the modified channel electrode. If we had moved closer to the areas of the PSD closest to the electrode in question, the errors would have been inadmissible due to the influence of that signal in the calculation of the impact point (Figure 23).

After verifying the need for calibration and the effect of asymmetries between amplifier channels due to external components and even the PSD and amplifiers, we proceeded to perform tests with temperature. However, although we attempted to heat the elements in one of the channels with a jet of hot air, the components of the different channels were very close and we were unable to measure variations due to temperature. As discussed in Section 3, this is because the temperature varies very little and it is very difficult to induce different temperature changes in such a small space.

### 5.2. Analysis of the Influence of Signal Noise

**Figure 24:**
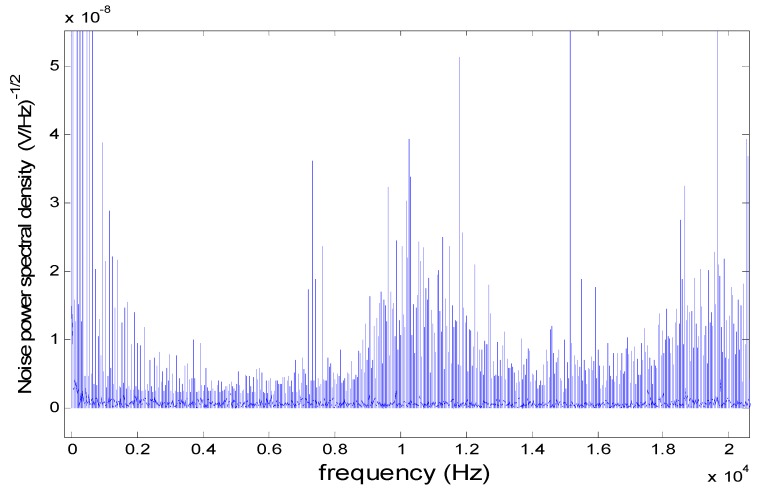
Spectral noise measured when the PSD sensor was not illuminated (image corresponds to channel 1).

To empirically assess the influence of SNR and measure the error in calculation of the point of incidence of the light beam on the PSD sensor surface, we first measured the spectral noise density of the system, using the channel 1 distribution shown in Figure 24. To obtain this information, we covered the PSD receiving surface and digitised the signals obtained by the different channels (f_s_ = 125 kHz and 12 bit resolution, with a SPAN of 0.1 V). From this data, we calculated the FFT, obtaining the results shown in Figure 24.

After calculating system noise, several tests were performed in 5 different PSD positions and at 3 SNR levels (40, 50 and 60 dB). For this, we measured the total noise on a bandwidth of 1 kHz centred on the frequency (8 kHz). The noise value was used to adjust transmission power to obtain an SNR of 60 dB. For tests of 50 dB and 40 dB, optical filters were applied until achieving a sensor signal strength corresponding to these SNR levels. For these measurements, we used 18 ADC bits to avoid being affected by quantization errors.

**Figure 25:**
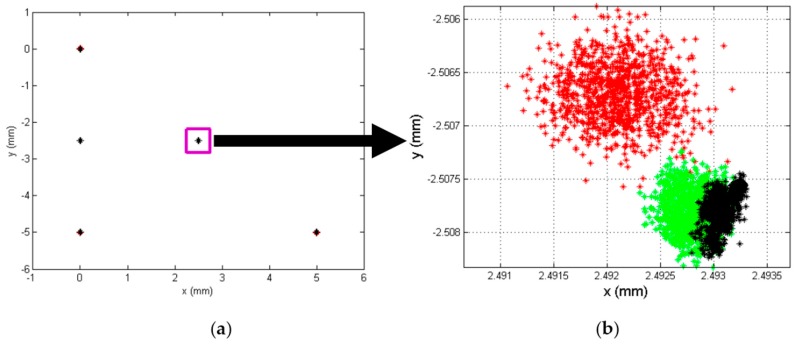
(**a**) Error due to noise for a SNR at 40 dB (red), 50 dB (green) and 60 dB (black).; (**b**) Enlarged of the (2.5,−2.5) point.

Table 3 shows a comparison of the data obtained from the experiments and the simulations, while Figure 25 shows the points obtained in the experimental tests, which can be compared with those shown in Figure 13 (simulation).

### 5.3. Analysis of the Influence of Quantisation Noise

**Figure 26:**
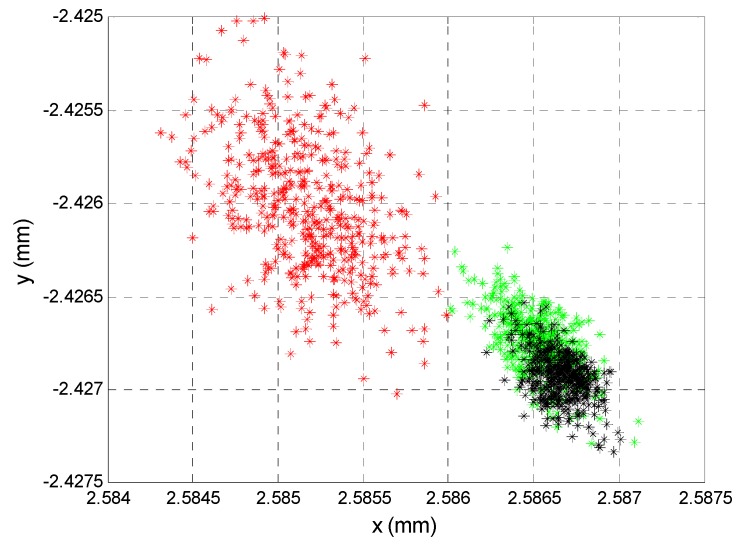
Error due to quantization noise with 6 bits (39 dB, red), 8 bits (51 dB, green) and 10 bits (61 dB, black).

To test the effect of ADC quantization noise, we used a signal that provided a value of 90 dB of system SNR, with a bandwidth of 1 kHz centred on the working frequency (8 kHz), as previously. In this case, the experiment was repeated with the same criteria as in all previous tests, but considering conversions with 6, 8 and 10 bits, to study how quantisation error affected determination of the PSD point. The results are depicted in Figure 26, where the position obtained with 6 bits (about 39 dB) is shown in red, with 8 bits (51 dB) in green and with 10 bits (61 dB) in black.

The averge, maximum errors and standard deviation for the three values of *SNR_Q_* are shown numerically in Table 4. Compared with Table 3, it can be seen that the dimensions were approximately the same. Thus, in order to eliminate any effect it would be sufficient to select the number of conversion bits with a *SNR_Q_* 20 times higher than system SNR. The values of SNR_Q_ and SNR are different because in the case of SNR_Q_ we only have been able to change the ADC bits and these values were the nearest to the SNR.

Neither the system nor quantitation SNR of the LPS system described here can be disregarded; however, it would be sufficient to ensure that the SNR > 40 dB in order to obtain a mean localization error of 0.27 mm at 5 m with commonly used optical systems. Furthermore, if the SNR were increased to 60 dB, the error at 5 m would be reduced to 0.04 mm.

## 6. Conclusions and Future Research

This paper reports a thorough analytical study of the factors affecting the localization errors obtained when using a PSD sensor and conditioning circuit. Our analysis revealed several types of errors, which can be classified as random and systematic. We found that systematic errors was a source of considerable error which cannot be disregarded and must be eliminated. Since this error is systematic, we have proposed a system model and calibration method as the basis for obtaining the model performance parameters and correcting the errors generated in the different sensor system channels. The most important aspect was the internal performance of the PSD itself and the system component tolerances, and we have proposed joint calibration. The results show that the proposed model and method are feasible to implement and use without the need for complex and costly infrastructures, and permit error mitigation. Although errors in determining the point of incidence are significantly attenuated when high quality components are used, calibration is still necessary due to the internal performance of the PSD and to avoid the propagation of errors to the next phase of geometric calibration, as they would be magnified in this stage.

Systematic errors are also caused by the effect of temperature coefficients. In this case, it may be sufficient to be careful with component placement in the printed circuit board design, placing them close together in areas that receive the same level of cooling so that they are not exposed to different surrounding temperatures or variations in temperature. In line with the conclusions presented here, we attempted to measure the influence of this but observed no differences, indicating that this error is resolved by following these recommendations.

Random errors cannot be calibrated, but as we have seen, the effect of these is negligible provided that certain criteria are observed when selecting components and their characteristics. For system SNR for localization at a distance of 5 m, it would be sufficient to ensure that the SNR > 40 dB in order to obtain a mean localisation error of 0.27 mm at 5 m with commonly used optical systems. Furthermore, if the SNR were increased to 60 dB, the error at 5 m would be reduced to 0.04 mm. These values could be obtained using currently available commercial transmitters due to the low working frequencies. Where this is not possible, multi-transmitter systems could be used since at these frequencies, the effects of different response delays and jitter are negligible. Likewise, quantitation errors have been shown to be negligible if the number of bits used is such that the SNR_Q_ is 20 dB greater than the SNR.

## Figures and Tables

**Table 1:** Maximum gain factor errors for the range of frequencies given in Figure 5.

	ΔR_f_ = 1 kΩ	ΔR_f_ = −1 kΩ	ΔR_f_ = −1 kΩ	ΔR_f_ = 1 kΩ
	ΔC_f_ = 15 pF	ΔC_f_ = 15 pF	ΔC_f_ = −15 pF	ΔC_f_ = −15 pF
Maximum error frequency (kHz)	13.793	18.038	13.793	18.038
Nominal gain to the maximum error frequency (k_nom_)	53,572	46,874	53,572	46,874
Maximum variation of the gain factor (Δk_o_)	4017.9	3515.6	−4017.9	−3515.6
Maximum variation of the gain factor (%)	7.5%			

**Table 2:** Standard deviation, average and maximum and position errors (2.5, −2.5).

SNR (dB)	20	30	40	50	60	70	80	90
Standard deviation (mm)	5.96 × 10^−3^	2.03 × 10^−3^	6.57 × 10^−4^	2.02 × 10^−4^	6.46 × 10^−5^	2.10 × 10^−5^	6.67 × 10^−6^	2.02 × 10^−6^
Average error (mm)	6.64 × 10^−2^	8.87 × 10^−3^	6.56 × 10^−4^	2.83 × 10^−4^	6.39 × 10^−5^	6.66 × 10^−5^	4.85 × 10^−6^	2.65 × 10^−6^
Max. Error (mm)	2.24 × 10^−2^	6.68 × 10^−3^	2.29 × 10^−3^	5.99 × 10^−4^	2.12 × 10^−4^	6.73 × 10^−5^	1.96 × 10^−5^	6.62 × 10^−6^

**Table 3:** Comparison between simulated and measured errors.

SNR (dB)	40		50		60	
	Measured	Simulated	Measured	Simulated	Measured	Simulated
Standard deviation (mm)	3.42 × 10^−4^	6.57 × 10^−4^	1.89 × 10^−4^	2.02 × 10^−4^	5.02 × 10^−5^	6.46 × 10^−5^
Average error (mm)	4.68 × 10^−4^	6.56 × 10^−4^	2.58 × 10^−4^	2.83 × 10^−4^	7.43 × 10^−5^	6.39 × 10^−5^
Max Error (mm)	1.06 × 10^−3^	2.29 × 10^−3^	5.68 × 10^−4^	5.99 × 10^−4^	9.51 × 10^−5^	2.12 × 10^−4^

**Table 4:** Errors due to quantization noise, and comparison with measured errors due to noise.

SNR (dB)	SNR_Q_ 39	SNR 40	SNR_Q_ 51	SNR 50	SNR_Q_ 61	SNR 60
Standard deviation (mm)	3.78 × 10^−4^	3.42 × 10^−4^	1.54 × 10^−4^	1.89 × 10^−4^	9.10 × 10^−5^	5.02 × 10^−5^
Average error (mm)	6.86 × 10^−4^	6.56 × 10^−4^	3.17 × 10^−4^	2.58 × 10^−4^	8.93 × 10^−5^	7.43 × 10^−5^
Max Error (mm)	2.72 × 10^−3^	1.06 × 10^−3^	9.10 × 10^−4^	5.68 × 10^−4^	5.20 × 10^−4^	9.51 × 10^−5^
